# Ancient origin and dynamic evolution of bivalent spider toxins

**DOI:** 10.1093/molbev/msag076

**Published:** 2026-03-24

**Authors:** Robin A Araya, Marius F Maurstad, David T Wilson, Lachlan D Rash, Mehdi Mobli, Kjetill S Jakobsen, Eivind A B Undheim

**Affiliations:** Centre for Ecological and Evolutionary Synthesis, Department of Biosciences, University of Oslo, Oslo 0316, Norway; Centre for Ecological and Evolutionary Synthesis, Department of Biosciences, University of Oslo, Oslo 0316, Norway; Advanced Analytical Centre, James Cook University, Smithfield, QLD 4878, Australia; School of Biomedical Sciences, The University of Queensland, St Lucia, QLD 4072, Australia; Australian Institute for Bioengineering and Nanotechnology, The University of Queensland, St Lucia, QLD 4072, Australia; Centre for Ecological and Evolutionary Synthesis, Department of Biosciences, University of Oslo, Oslo 0316, Norway; Centre for Ecological and Evolutionary Synthesis, Department of Biosciences, University of Oslo, Oslo 0316, Norway

**Keywords:** funnel-web spider, venom, multi-domain peptide, inhibitory cystine knot, DkTx, pi-hexatoxin

## Abstract

Bivalent peptide toxins comprising 2 cysteine-rich domains have evolved from single-domain precursors on multiple occasions in animal venoms, resulting in enhanced molecular target selectivity and avidity. Although bivalent toxins are emerging as prevalent in animal venoms, the genomic and evolutionary processes driving the transitions between single- and multi-domain architectures remain poorly understood. Here, we investigated the evolution of bivalent inhibitor cystine knot (ICK) toxins in spider venom. We first generated a genome assembly of the tree-dwelling funnel-web spider *Hadronyche cerberea*, revealing a massive expansion of ICK toxin-encoding genes, including the bivalent *π*-hexatoxin-Hc1a. All ICK toxin genes share a conserved 3-exon structure, flanked by transposable elements (TEs) that may have facilitated gene expansion. This gene structure is shared by the Hc1a subfamily, where the entire mature bivalent toxin is encoded by the third exon. Leveraging de novo transcriptome assemblies from 86 spider species along with venom proteomic data, we show that bivalency in the Hc1a subfamily is of ancient origin and evolved via intra-exonic duplication not involving introns. This was followed by domain expansion and recurrent domain losses mediated by point mutations, deletions, and unequal crossing-over facilitated by high interdomain sequence similarity. In contrast, the bivalent toxin DkTx from *Cyriopagopus schmidti* is confined to a small group of tarantulas, where it appears to have evolved once, with subsequent domain losses potentially linked to TE activity. Our findings reveal that singular events of domain duplication can give rise to complex, asymmetrical evolutionary trajectories shaped by gene instability and selective retention of functional domains.

## Introduction

Bivalency is a prevalent natural mechanism for improving the potency and selectivity of intermolecular interactions. Most commonly known for augmenting the avidity of antibodies through a dimeric binding mode, bivalency can drastically enhance the biological effect of biomolecules (Mammen et al. 1998; Bohlen et al. 2010; Chassagnon et al. 2017; Maxwell et al. 2023). Several bivalent peptide toxins have recently been described in various animal venoms that show a remarkable avidity for their target receptors (Bohlen et al. 2010; Chassagnon et al. 2017; Maxwell et al. 2023). These bivalent toxins consist of 2 stable cysteine-rich domains that are connected by a short peptide linker, ultimately mimicking the structural properties of antibodies while retaining a molecular size in the range of peptides (≤∼10 kDa). These properties, along with high selectivity and potency that is often associated with toxins from animal venoms (eg Muttenthaler et al. 2021), have sparked a growing interest in bivalency for novel drug development targeting ion-channel-related conditions (eg Redd et al. 2021).

Despite the growing efforts in studying bivalent peptide toxins, little is known about how they evolved. Protein repeats are commonly believed to evolve through exon shuffling involving introns (Björklund et al. 2006; Moore et al. 2008). In this case, the exons involved encode full-length protein domains—ie a self-stabilizing and functional unit of the protein—duplicated through misalignment and nonallelic homologous recombination (NAHR) facilitated by introns (Björklund et al. 2006; Moore et al. 2008). The rate of domain repeat expansion tends to increase with the number of repeating units (Looman et al. 2002), leading to long tandem-duplications of domains, which can be coexpressed as multivalent proteins through bypassing of stop codons or post-translational conjugation of domains. Although introns seem to drive the formation of protein repeats, this is not reported for the bivalent toxins described to date (eg Sachkova et al. 2014). In fact, the toxin-encoding genes of the Australian funnel-web spider *Hadronyche infensa,* the venom of which contains the bivalent acid-sensing ion-channel (ASIC) inhibitor π-Hexatoxin Hi1a (henceforth just Hi1a), are believed to lack introns altogether, questioning the role of exon shuffling in generating bivalent peptide repeats (Pineda et al. 2012). However, the availability of genomic data for venomous lineages has so far been scarce, limiting our ability to thoroughly investigate the features of bivalent toxin-encoding genes.

Toxins in animal venoms generally evolve through duplication and neofunctionalization of house-keeping genes (eg enzymes and signaling molecules) (Casewell et al. 2013). These toxin gene families often rapidly evolve new functionalities through gene duplication and accumulation of mutations thanks to the structural robustness conferred by their disulfide-rich core domain (Sunagar et al. 2013; Undheim et al. 2016). This variability is exemplified by the expansion of neurotoxins containing the inhibitor cystine knot (ICK) fold in different spider venoms (Zhu et al. 2003; Vassilevski et al. 2009; Pineda et al. 2020) with as many as 13 categorized superfamilies of ICK toxins in 1 single species (*H. infensa*) (Pineda et al. 2014, 2020). ICK toxins are particularly predominant in the venoms of mygalomorph spiders, from which 2 of the 4 bivalent neurotoxins characterized in detail have been identified (Liu et al. 2023; Maxwell et al. 2023): Hi1a from *H. infensa* (Chassagnon et al. 2017) and a capsaicin receptor (TRPV1) agonist named Tau-theraphotoxin Hs1a, commonly referred to as double-knot toxin (henceforth just DkTx), from the Earth tiger tarantula *Cyriopagopus schmidti* (Bohlen et al. 2010). However, while these double-ICK neurotoxins are hypothesized to be the results of recent domain duplications, their evolutionary histories and taxonomic prevalence remain unknown. This both limits our understanding of the mechanisms underlying such evolutionary innovation and hinders further discoveries of multi-domain neurotoxins with therapeutic potential.

Here, we investigated the evolutionary histories and underlying genomic mechanisms of the Hi1a-like and DkTx-like toxin families. Leveraging a long-read-based (HiFi) genome assembly of a single *Hadronyche cerberea* individual, we found that ICK-encoding genes have undergone a large expansion possibly facilitated by transposable elements (TEs) and that these genes, including the bivalent π-Hexatoxin Hc1a (henceforth just Hc1a), share a conserved 3-exon gene structure. Using proteotranscriptomic evidence, we further show that bivalency in the Hc1a family is the result of a single ancient domain duplication, and that this event was followed by multiple domain reversals across distantly related families of the mygalomorph spiders. We compared this dynamic pattern to the evolution of the bivalent tarantula toxin DkTx and show that it represents a relatively recent and independently evolved family of double-ICK toxins. We also found evidence of multiple domain loss events in this family, although these likely occurred through a different molecular mechanism than the Hc1a family.

## Results

### Gene expansion and conserved architecture of ICK-encoding toxins

**Figure 1:**
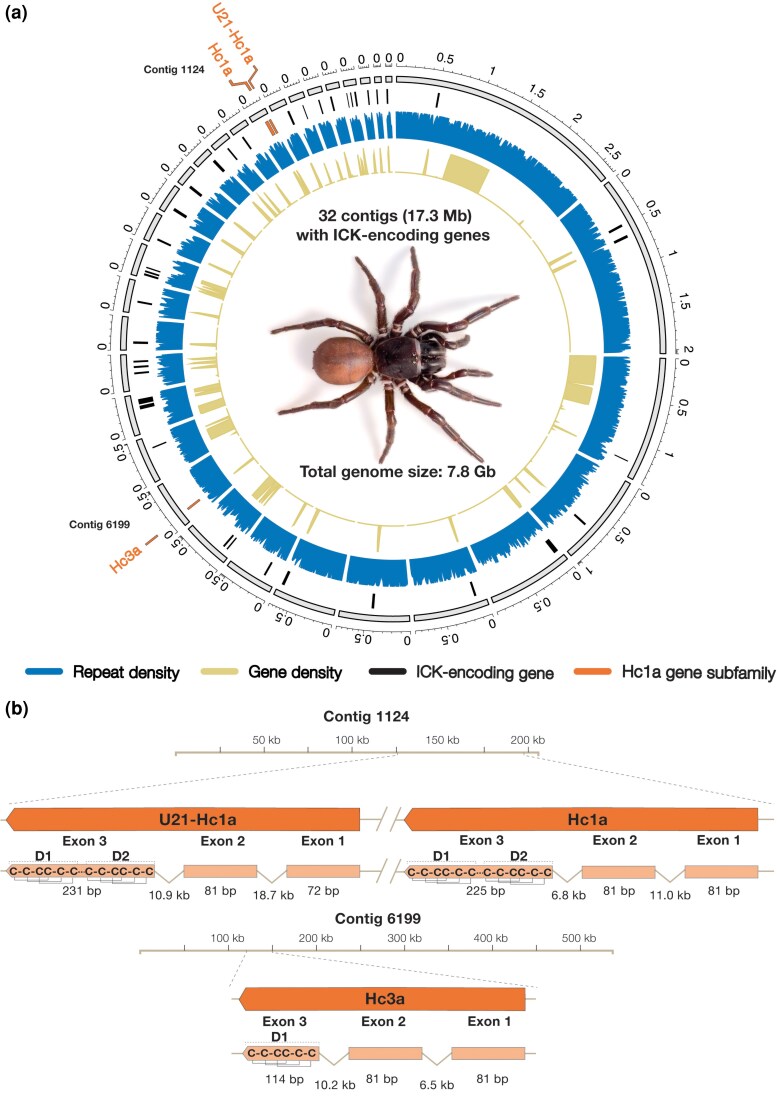
Overview of ICK-encoding genes in *H. cerberea*. a) Circos-plot showing the contigs harboring ICK-encoding genes in the genome assembly of *H. cerberea*. The rings from inner to outer display the gene density (all genes; yellow, filled), repeat density (blue, filled), ICK-encoding gene position (black bars), and position along each contig in Mb (gray). Repeat density was plotted within nonoverlapping sliding windows of 10 kb. Genes of the Hc1a subfamily are highlighted in orange along their respective contig number. b) Gene positions and structures of the Hc1a subfamily. Positions are shown along the contig tracks in kilobases. Full-genes insets from the contig tracks are shown in orange (top), highlighting the 3 exons below, (from right) representing signal- (exon 1), pro- (exon 2), and mature-peptides with the typical ICK pattern (exon 3), with connective lines above the third exons displaying disulfide bonds between cysteines and domains of the bivalent variants labeled as D1 and D2. Exons and introns are not drawn to scale, and actual sizes are displayed below the genes.

We generated a de novo 13X HiFi genome assembly of a female *H. cerberea*. We estimated a primary assembly size of 7.8 Gb (N50 = 420 kb; Figure S1 and Table S1a), which is expected for this group of mygalomorph spiders (eg You et al. 2024). While the assembly is spread across 35,311 contigs after contaminant removal (Figure S1), gene completeness was estimated to 92.3% (using the Arachnida Benchmarking Universal Single-Copy Orthologs (BUSCO) gene set containing 2,934 genes; Figure S1 and Table S1b). Using transcriptomic data from the same *H. cerberea* individual in combination with publicly available data (see *Materials and Methods*), we annotated a total of 14,761 protein-coding genes with BRAKER3 (Gabriel et al. 2024). This is lower than detected in other mygalomorph spiders (eg 39,687 in *Macrothele yani*), but with a low proportion of duplicated genes (see full comparison of assembly statistics between available assemblies from Mygalomorphae and Mesothelae in Table S1c). Among these genes, we identified a large expansion of ICK-encoding genes (ICK genes) comprising 42 paralogs (Table S2) dispersed across 32 different contigs (Fig. 1a). Of these 42 ICK genes, we identified 4 close paralogs in *M. yani* (out of 12 ICKs identified in You et al. 2024, of which one is a bivalent ICK), 2 in its sister species *M. cretica*, 3 in *Aptostichus stephencolberti* (family: Euctenizidae), 1 and 2 distinct paralogs in the 2 theraphosidae species *Chaetopelma lymberakisi* and *Pterinocilus murinus*, respectively, 1 in an Atypus species (family: Atypidae) and 2 in *Ryuthela nishihirai* (Mesothelae), the latter being the sister order to all spiders (see *Materials and Methods*).

Following gene annotation, we clustered the amino acid sequences encoded by the ICK gene set into putative subfamilies using CLANS (Frickey and Lupas 2004) and nucleotide sequences with MMseqs2 (Steinegger and Söding 2017) (Figure S2). This gene clustering showed that the bivalent double-ICK toxin Hc1a forms a group with 2 other paralogs: a bivalent toxin named U21-Hexatoxin-Hc1a (henceforth just U21-Hc1a) that is a near-identical ortholog of Hi1c from *H. infensa* (Chassagnon et al. 2017), and *π*-Hexatoxin-Hc3a (henceforth just Hc3a), which is characterized as a single-ICK toxin with sequence similarity to both of the domains of Hc1a (Budusan et al. 2024). Of these, U21-Hc1a is a gene duplication upstream of Hc1a (Fig. 1b, see clustering in Figure S2); whereas, Hc3a is placed on a different contig with no other nearby paralogs (Fig. 1b). We did not find any potential ancestral genes encoding only one of the domains of Hc1a, implying that the ancestral peptide precursor of Hc1a was not retained as a single-domain peptide after domain duplication.

To identify which of the identified ICK genes encode putative toxins, we next examined their differential expression across the venom glands, chelicerae, prosoma, opisthosoma and legs of the same specimen. We found that 36 were putative toxins that are differentially expressed in the venom glands, whereas the remaining 9 genes show near-zero expression in the venom glands and higher expression in different tissue (Figure S3). Of these, Hc1a and its paralogs, U21-Hc1a and Hc3a, are exclusively expressed in the venom glands, with negligible expression in other tissues (Figure S3). Analyzing crude venom by shotgun proteomics further supported the presence of at least 24 ICK paralogs in the venom (Table S2), all of which show high expression in the venom glands (14/24 exclusively expressed in venom glands; 10/24 additionally show low expression in the chelicerae, possibly due to minor contamination during dissection). We identified several unique tryptic fragments belonging to Hc1a and Hc3a, as well as fragments covering parts of U21-Hc1a (Table S2 and Figure S4). Taken together, these results show the Hc1a subfamily is solely utilized in the venom of *H. cerberea*, although U21-Hc1a may only be a minor venom component.

Manually investigating the 42 ICK genes in *H. cerberea*, we detected a universally conserved 3-exon gene structure (Fig. 1b). Contrary to previous findings that suggest most hexatoxins are encoded by intronless genes (Pineda et al. 2012), we found that every ICK-encoding gene was separated by introns, where the first 2 exons (signal- and propeptide) were separated by a phase 1 intron and the last 2 exons (propeptide and mature peptide) by a phase 2 intron (Table S2). Importantly, we found that the mature peptides, including the bivalent Hc1a-like peptides, are in their entirety encoded by the last exon (exon 3; Fig. 1b). The absence of intronic separation between the 2 domains suggests that the ancestral Hi1a-like peptide evolved bivalency through intra-exon duplication. This scenario is also supported by the sequence homology between the short peptide linker and the last 4 residues of the C-terminal domain (VPIS vs. APIT), which indicates that the linker is derived from the C-terminal tail of the ancestral single-domain (Figure S5).

Of the full-length ICK paralogs identified in the other genome assemblies from Mygalomorphae and Mesothelae (Table S1c), we found that they shared the same conserved 3-exon structure as in *H. cerberea*. However, the orthologs of the 2 ICK genes identified in *R. nishihirai* are not expressed in the venom glands of *H. cerberea*—only in the prosoma and opisthosoma—suggesting that they do not encode toxins. One of these genes (g14427 in *H. cerberea*) was also detected as conserved orthologs in all spider genomes examined here, highlighting this orthogroup as a potential candidate for the ancestral nontoxin ICK clade that gave rise to spider venom ICK toxins.

### TEs likely facilitated ICK gene expansion but not domain duplication

**Figure 2:**
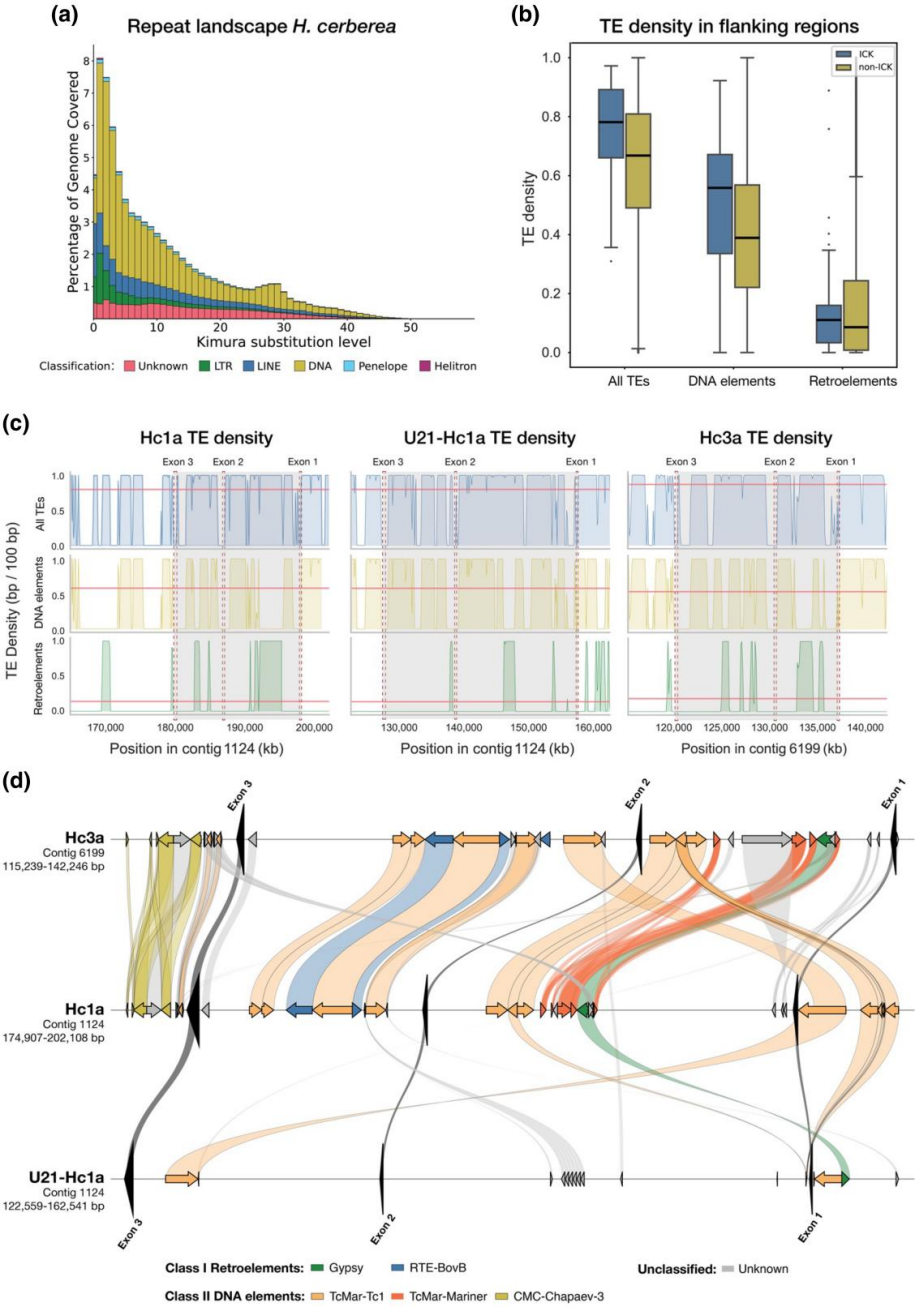
Repeat landscape and TE density in the Hc1a subfamily. a) Repeat landscape in the *H. cerberea* genome assembly, showing the fraction (% genomic coverage) of TE orders plotted against the Kimura divergence (%) to their consensus. The bins are colored according to the legend. b) Boxplot of TE density (%) only within flanking regions (+/− 5,000 bp of gene boundaries) of ICK genes versus non-ICK genes in *H. cerberea*. TE classes are colored according to legend. c) TE density within the genes (+/− 5,000 bp) of the Hc1a subfamily, plotted within nonoverlapping sliding windows of 100 bp for all TEs (top, blue), DNA elements (middle, yellow), and retroelements (bottom, green). Mean TE density on the same contig are shown as red horizontal lines. Genes are highlighted in light gray and exon boundaries are shown in dashed red lines. d) TE insertions that are shared between at least 2 genes of the Hc1a subfamily. Positions of each insertion are shown as arrows along the gene tracks for Hc3a (top), Hc1a (middle), and U21-Hc1a (bottom). Putative syntenic insertions belonging to the same TE family are connected with filled, shaded lines. Exons are highlighted as black arrows. TE superfamilies are colored according to the legend.

To further explore the structure and genomic background of the Hc1a-like genes, we investigated whether these and other ICK genes were associated with insertions of repetitive DNA. We created a species-specific, manually curated library of TEs for *H. cerberea* (Table S3). The final library masked 72.8% of the *H. cerberea* assembly, where 71.9% represented interspersed repeats (Fig. 2a; Table S4). Of these, 42.6% were class II DNA elements, with an astonishing 31.5% representing one superfamily of TEs (Tc1/Mariner), whereas 12.4% were long interspersed nuclear elements and 7.0% were long terminal repeat (LTR) retrotransposons. Moreover, we find that a large portion of the annotated TEs share a low divergence to their consensus sequences, implying that there are many active TEs that participate in shaping the genome of *H. cerberea*.

Interestingly, the flanking regions of ICK genes (+/− 5,000 bp of gene boundaries) show high TE density when compared with non-ICK genes (Fig. 2b; see *Materials and Methods*). This increase around ICK genes is particularly prominent in the class II DNA elements, whereas the amount of flanking class I retroelements were comparably low around both ICK and non-ICK genes (Fig. 2b). Moreover, contigs harboring ICK genes had higher TE content (80.32%) compared to both the overall assembly and contigs containing only non-ICK genes (72.66%) (Figure S6). In particular, we observed an increase in DNA elements with a moderate degree of divergence to its consensus (>10%) on the contigs with ICK genes, suggesting an association between old DNA elements and the expansion of ICK-encoding genes. To explore the relationship between TEs and bivalent ICK genes, we plotted the TE density spanning the Hc1a subfamily (gene region +/− flanking regions of 5,000 bp), using nonoverlapping sliding windows of 100 bp (Fig. 2c). A common feature of these genes is the presence of long introns separating short exons. Our TE density plots displayed an above-average TE density within the introns and flanking sequences, compared with the overall TE density on the same contig, primarily consisting of DNA elements (Fig. 2c). However, we did not find TEs that overlapped any exon borders.

Given the lack of introns separating the double-ICK domains (exon 3) in Hc1a and U21-Hc1a, we assessed whether the domain duplication in these genes could have been facilitated by the presence of similar TEs flanking the third exon in the different Hc1a-paralogs (ie Hc1a, U21-Hc1a, and Hc3a). By filtering on TE family insertions that were shared by the 3 Hc1a-like genes, we found no similar TEs immediately flanking exon 3 in any of the 3 genes (Fig. 2d). Of the few TE families that were shared across all 3 Hc1a-like genes (introns and flanking regions), the insertions had a mean length of 225 bp (17 TE insertions; SD 180 bp). However, when comparing TEs shared by only 2 of the Hc1a-like genes, and not all 3 genes, we found multiple conserved insertions of class II DNA elements, particularly of the TcMariner-Tc1 superfamily (Fig. 2d). Comparison of these insertions at the level of TE families showed that Hc1a shares half of its TE insertions with the monovalent Hc3a (22 shared TE families of total 44 distinct TE families within the gene region), indicating that Hc1a and Hc3a are closely related. In contrast, U21-Hc1a harbored 7 TE families that were shared with either Hc1a or Hc3a, out of a total 61 different TE families within its gene region (Fig. 2d). These findings suggest that while TEs might have been involved in the expansion of the ICK toxin gene family, they probably did not facilitate the intra-exon duplication that led to bivalency in the Hc1a subfamily.

### Bivalency in Hc1a evolved once from an ancient domain duplication

**Figure 3:**
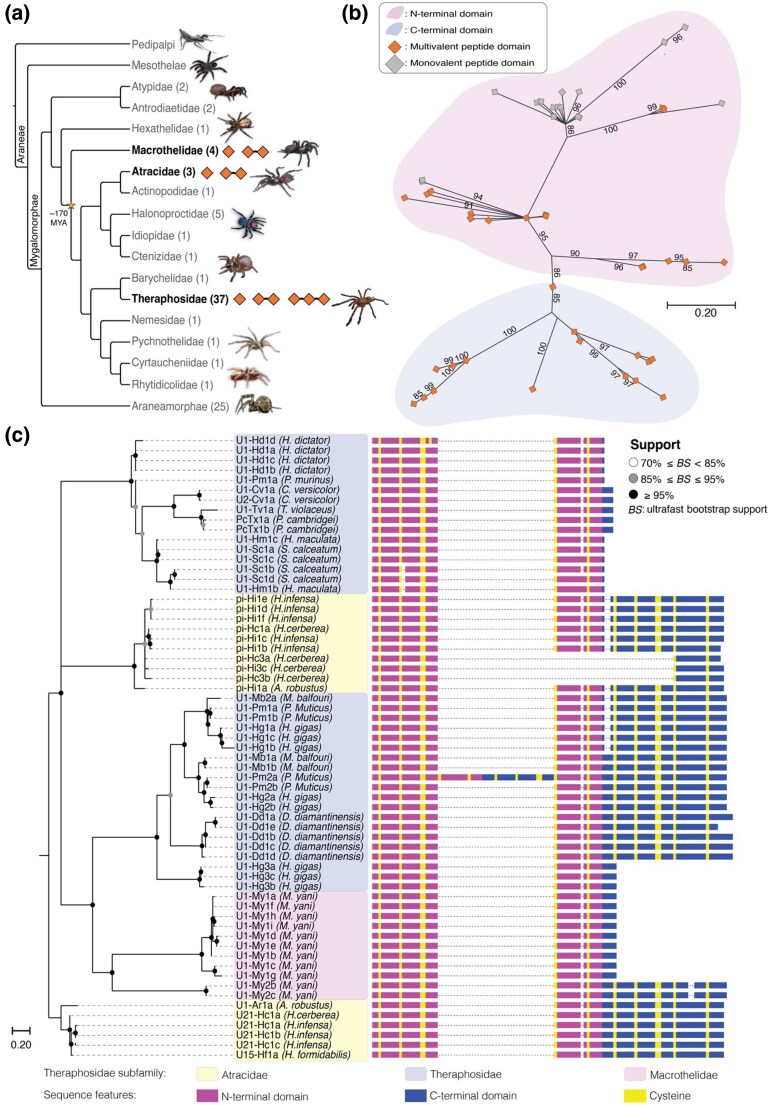
Evolutionary origins of Hc1a-like peptide variants. a) Distribution of Hc1a-like orthologs across Mygalomorphae, only showing families included in our Aranea RNA database (number of species shown in parentheses). Peptides are annotated as either single-, double- or triple-ICKs, shown as orange squares. Phylogeny adapted from Opatova et al. (2020), with representative species from selected families along the tree shown on the right. b) Domain ML phylogeny for the N- and C-terminal domains, including single-domain peptides of Hc1a orthologs. Peptides clustering as N-terminal domains are colored in light pink (top), C-terminal peptides are colored in light blue (bottom). Domains from multivalent peptides are colored orange, monovalent peptides are colored in gray. Bootstrap values are shown as numbers. c) ML phylogenetic tree of identified homologous Hc1a-like peptides. The tree topology was based on prepropeptide sequences (signal-peptide and pro-peptide regions), and the alignment on the right shows the functional ICK domains that best represents the data presented in our combined phylogenetic analyses. Aligned domains are colored according to similarity to the N-terminal (pink) or C-terminal (blue) domain, highlighting the conserved cysteine frameworks (yellow). Putative toxin names correspond to Table S6 and Data S1, with species names in parentheses and colored according to family within Mygalomorphae. Bootstrap support values are shown as circles, colored as indicated in the inset. Species images and credits (from top): *Typopeltis crucifer* (CC0), *Liphistius malayanus* (Photo: dhfischer, CC BY-SA 4.0), *Sphodros rufipes* (CC0), *Hexathele* sp. (CC0), *Macrothele* sp. (Photo: nielwame, CC BY 4.0), *Atrax robustus* (Photo: Ethan Yeoman, CC BY-NC 4.0), *Missulena occatoria* (CC0), *Cteniza moggridgei* (Photo: Ludivine Lamare, CC BY 4.0), *Chilobrachys natanicharum* (own photo), *Calisoga longitarsis* (Photo: Taji, CC BY 4.0), *Fufius* sp. (CC0), *Parasteatoda tepidariorum* (CC0).

Given that all mature ICK peptides are encoded by a single exon and that we find no evidence of alternative splicing, we next used transcriptomic data to obtain a denser taxonomic sampling for examining the evolution of Hc1a. We searched a custom sequence database for Hc1a homologs comprising 175 de novo transcriptome assemblies from 86 phylogenetically diverse species of Araneae (Table S5, see Figure S7 for an overview of available data). In addition, we searched for Hc1a homologs using online protein databases (see *Materials and Methods*). The resulting set of 67 putative Hc1a-homologs revealed that the bivalent Hc1a is exclusive to Mygalomorphae and evolved through a domain duplication predating the split between Macrothelidae and Atracidae (>150 MYA; see Opatova et al. 2020) (Fig. 3; Table S6a; Data S1). We did not find any association between sequencing depth, sex, or average read length of the databases used and the presence of bivalent Hc1a-like homologs (Figure S8). We also detected Hc1a-like peptides in multiple genera within Theraphosidae, but not in interleaving families, which also lacked single-domain Hc1a-like orthologs (Fig. 3). However, all spider families with bivalent Hc1a orthologs also contained multiple instances of deviation from bivalency, including several single-domain variants as well as an expansion to a trivalent Hc1a-like toxin in the theraphosidae *Pelinobius muticus* (U1-Pm2A; Fig. 3a). We also found high conservation of the peptide linker sequences, with the first 3 linker residues (VPI) being fully conserved across all multivalent entries and the latter 3-5 residues being comparably more variable, albeit with high sequence similarity (Figure S9a).

Utilizing the 2 near-chromosome level genome assemblies from Theraphosidae (*C. lymberakisi* and *P. murinus*) and one from the interleaving family Euctenizidae (*A. stephencolberti*, Table S1c; see Figure S7 for phylogenetic placement), we mapped the 67 different Hc1a-like peptides to identify potential gene and pseudogene regions (see *Materials and Methods*). We were able to locate the gene encoding the monovalent Hc1a-like peptide in *P. murinus* (U1-Pm1a) and a potential pseudogene region in *C. lymberakisi,* which mapped to parts of the mature Hc1a peptide (Figure S10). However, we were unable to locate any candidate regions in *A. stephencolberti*.

To examine the origin of bivalency in Hc1a, we first analyzed the individual domains of the 67 Hc1a homologs. We partitioned the N- and C-terminal domains of the multivalent sequences and aligned these domains with the single-domain Hc1a variants to create a maximum likelihood (ML) phylogenetic tree of all ICK domains (Fig. 3b). The resulting domain phylogeny displayed a clear distinction between the C- and N-terminal domains separating into 2 well-defined clades, where the N-terminal domains clustered together with the single-ICK toxin variants (Fig. 3b), suggesting a single ancient domain duplication within this gene family.

**Figure 4:**
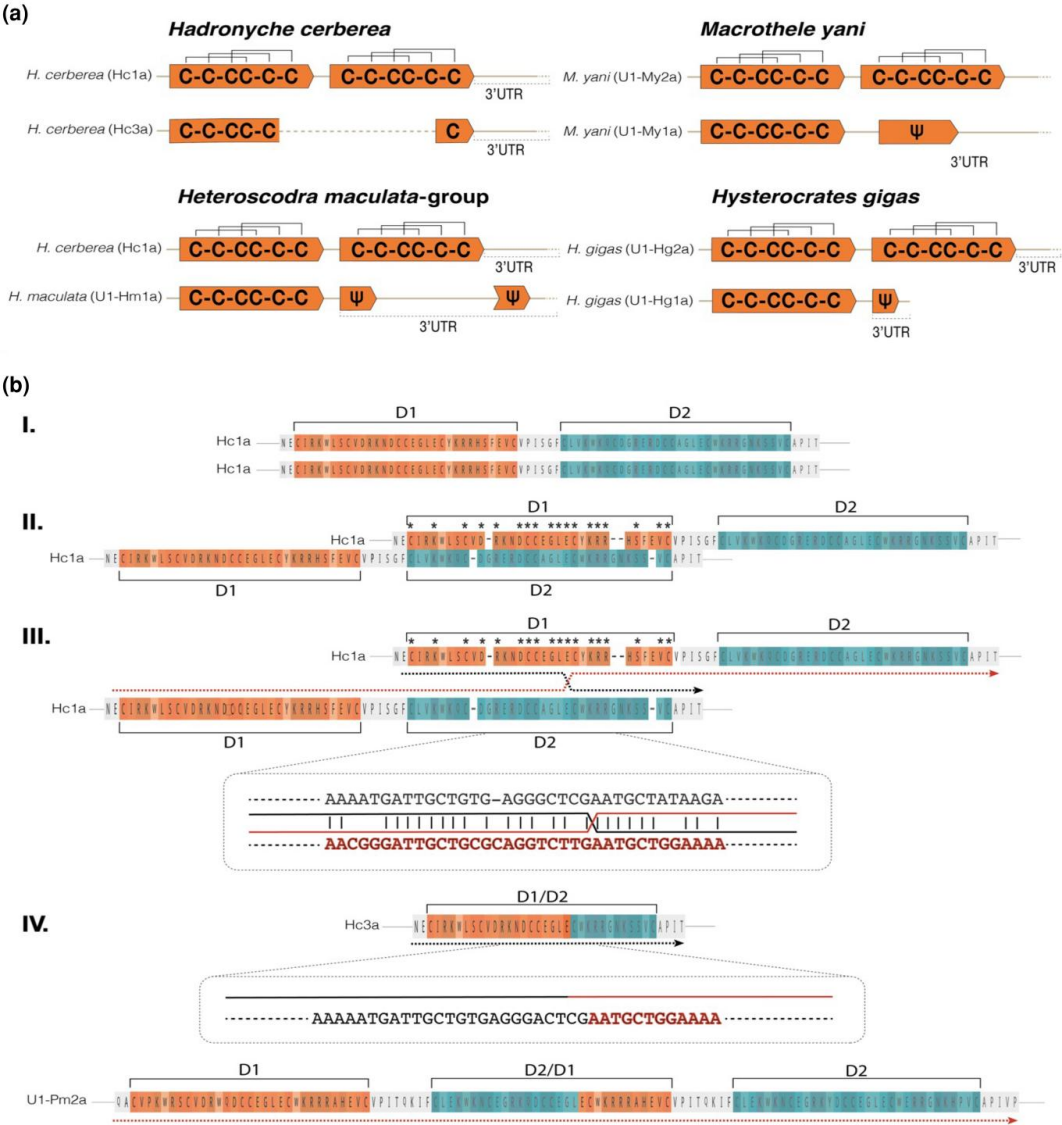
Molecular processes of domain loss and gain in Hc1a-like peptides. a) Schematic illustration of representative monovalent Hc1a-like peptides in relation to their closest bivalent ancestor. Domains are shown in thick arrows (orange) with proposed cysteine framework, and pseudogenes in 3′UTRs are labeled with Ψ. b) Proposed sequential pathway of the evolution from the bivalent Hc1a to a monovalent (Hc3a) and trivalent (U1-Pm2a) peptide product. The N- and C-terminal domains are labeled D1 and D2 and colored in orange and teal, respectively, with color nuances representing amino acid properties. The proposed regions facilitating unequal crossing-over are zoomed in, displaying the nucleotide sequences from D1 (black) and D2 (red), and sequence identity as vertical bars and nucleotide strand identity as black (D1) or red (D2). Strand direction is shown below the peptides as arrows colored according to strand origin.

We next used the prepropeptide sequences from the Hc1a-like homologs to generate a ML phylogeny representing the evolutionary trajectories of full-length Hc1a-like peptides. The resulting phylogeny unveiled a pattern suggesting one single event of domain duplication followed by multiple independent domain losses across different taxa, supporting a hypothesis of a single origin of bivalency in the Hc1a subfamily (Fig. 3c; see Figures S11a and S12a for full-peptide data). Importantly, the early evolution of bivalency in the Hc1a subfamily is robust to alternative tree topologies with different rooting (Figure S13), given the well-supported topology of the individual domains (Fig. 3b). Similarly, the number of independent domain losses is either 3 or 4 (Figure S13), depending on whether the tree is rooted with the exclusively single-domain clade of theraphosidae toxins (top clade, Fig. 3c) or the U21-Hc1a orthogroup (Figure S13). However, based on sequence divergence of U21-Hc1a and the potential presence of pseudogenized domain 2 in the single-domain clade (Fig. 4a; see below), we consider the topology presented in Fig. 3c the most likely. Taken together, these findings suggest an evolutionary scenario of a single, ancient domain duplication, followed by repeated domain losses (Fig. 3c): once in *H. cerberea* (Atracidae), once in *M. yani* (Macrothelidae), and on 2 separate occasions in Theraphosidae.

### Dynamic evolutionary history of multivalent Hc1a-like toxins

To elucidate the molecular evolutionary processes behind the recurrent reversals from bivalency to monovalency, we first looked for signs of domain loss through the acquisition of premature stop codons. We used Hidden Markov Models (HMMs) from Hc1a-like single-domain nucleotide sequences and peptide sequences to search for the presence of pseudogenes in the 3′UTRs (see *Materials and Methods*) that could represent remnants of the C-terminal domains in the monovalent sequences. We also combined HMM searches with mapping methods of bivalent and monovalent sequences that account for frameshifts (eg tblastx) and nucleotide sequence alignments. We detected a strong 32 bp signal for the presence of a C-terminal domain pseudogene in the 3′UTR of the monovalent U1-My1a group from *M. yani* (Fig. 4a), resembling the second domain in the bivalent U1-My2a (*M. yani*) (Figure S14a). Through visual inspection of sequence similarity within nonoverlapping 5 bp sliding windows of the (i) U1-My1a transcript (coding sequence and 3′UTR), (ii) the bivalent U1-My2a transcript and (iii) only the C-terminal (second) domain and proceeding 3′UTR of U1-My2a, we observed a clear sequence resemblance between the (i) 3′UTR of U1-My1a and the (ii) C-terminal domain of U1-My2a. Domain loss in U1-My1a is thus likely to have occurred through a point mutation (TGT → TGA), leading to a stop codon at position 126 bp (Figure S14a).

We performed similar analyses for the remaining 3 monovalent clusters. For the single ICK-toxin U1-Hg1a (*H. gigas*), we found a short segment in the partial, truncated 3′UTR resembling the C-terminal domains in the bivalent toxins U1-Hg2a and U1-Hg3a (*H. gigas*) (Fig. 4a; Figure S14b). The domain loss appears to have been driven by a 5 bp deletion in positions 126-130 bp, leaving a stop codon (TGA) at 124-126 bp. Within the group of monovalent toxins in Theraphosidae (named Hm1a group), we investigated the sequence most closely resembling the Hc1a nucleotide sequence, namely U1-Hm1a (*Heteroscodra maculata*). We found a low-scoring single-domain nHMM in the 3′UTR of U1-Hm1a within a region of moderate sequence similarity to Hc1a, suggestive of domain loss also in this group of monovalent peptides, supporting the topology presented in Fig. 3c. The domain loss can be ascribed a putative point mutation (GAA-TAA) in position 112 bp (Figure S14c).

Contrasting the above cases, the single-domain Hc3a in *H. cerberea* has evolved through a completely different mechanism. In line with a previous study of Hc3a (Budusan et al. 2024), peptide and nucleotide sequence alignments of Hc3a and its bivalent precursor Hc1a display the highest sequence similarities between the first half of Hc3a and the N-terminal domain of Hc1a, and its second half resembling the C-terminal Hc1a domain (Fig. 4; see also Budusan et al. 2024), supporting that Hc3a is a fusion product of the 2 domains of Hc1a. To explore the mechanism behind this apparent domain fusion, we aligned the 2 domains of Hc1a (peptide and nucleotide sequence alignments of N- and C-terminal domains), revealing that high interdomain sequence similarity, particularly prominent in a 25 bp stretch with 84% (21/25 bp) sequence identity, has led to the domain fusion through unequal crossing-over between the 2 Hc1a domains (Fig. 4b; Figure S14d). For comparison, we also self-dot-plotted the Hc1a coding sequence to evaluate whether the domain fusion could stem from a hairpin-induced indel formed by palindromic DNA sequences. However, we found no evidence of palindromic sequences (Figure S15), suggesting unequal crossing-over between the domains is by far the most likely scenario. The resulting monovalent product, Hc3a, mainly consists of the N-terminal domain of Hc1a, attributing its similar activity toward ASIC1a as Hc1a (Budusan et al. 2024).

Interestingly, the scenario of unequal crossing-over in Hc1a leading to domain reversal in Hc3a also leaves a trivalent peptide byproduct comprising the N- and C-terminal domains of Hc1a, separated by a third domain that is a fusion product of the 2 ancestral domains. While we did not detect signatures of such a product in the genome of *H. cerberea*, it provides a likely molecular mechanism for the origin of the putative triple-domain toxin detected in the venom gland transcriptome of *P. muticus* (U1-Pm2A; Fig. 4b). A triple-domain ICK peptide has, to our knowledge, not previously been described and represents an interesting variant of putative multivalent neurotoxins for further exploration.

### The bivalent tarantula toxin DkTx is confined to a small subgroup of Theraphosidae

**Figure 5:**
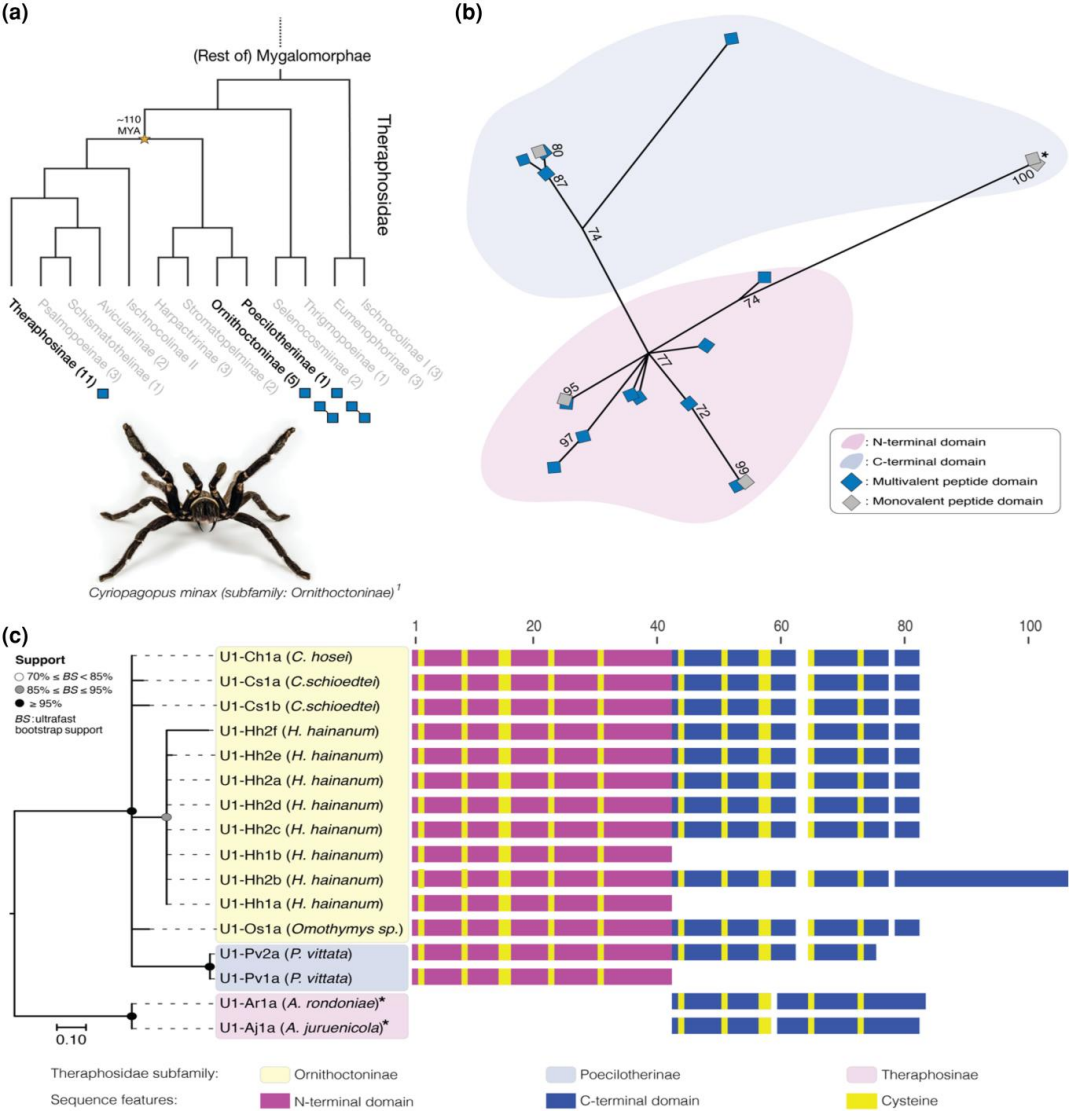
DkTx is confined to a small group of Theraphosidae. a) Phylogenetic distribution of DkTx-like peptides in Theraphosidae, highlighting the 3 subfamilies with monovalent and/or bivalent peptides shown as single or two connected diamonds, respectively. The number of species included in our Aranea transcriptome database are shown within parentheses. b) Domain ML phylogeny for the N- and C-terminal domains of DkTx, including single-domain peptides. Sequences clustering as N-terminal domains are colored in light pink (bottom), C-terminal sequences are colored in light blue (top). Domains from multivalent peptides are colored dark blue, monovalent peptides are colored in gray. Bootstrap values are shown as numbers. c) ML phylogeny for homologous peptides of DkTx with prepropeptides. Alignments are colored according to N-terminal (purple) or C-terminal similarity (blue), with cysteine residues shown in yellow. Positions (aa) are shown above the alignments. Peptides are labeled as in Table S7, with species names in parentheses and colors indicating subfamily. *Alignment and partitioning with C-terminal domain is based on nucleotide sequence similarity in Figure S14g. ^1^Original image of *Cyriopagopus minax* taken by Mako Pisces.

Given the ancient origin of bivalency in the Hc1a subfamily, we next examined the distribution and evolution of DkTx-like peptides—the other known bivalent ICK neurotoxins in mygalomorph spider venoms. Although there are no currently available genomic data for *C. schmidti* or any close relatives, we applied the same transcriptomic data mining-approach approach as with Hc1a under the assumption that the ICK gene structures are conserved. Screening for DkTx homologs in our Araneae database (which covers all but one subfamily of Theraphosidae) identified a total of 20 putatively homologous DkTx-like sequences (Table S6b). In contrast to Hc1a, we found that DkTx is confined to a small group of 2 sister subfamilies within Theraphosidae: Ornithoctoninae and Poecilotheriinae (Fig. 5). Their peptide linkers are nearly identical (>95% identity across the linker residues, Figure S9b). We also identified monovalent single-domain DkTx variants across Ornithoctoninae, Poecilotheriinae, and the distantly related genus *Acanthoscurria* (subfamily Theraphosinae) (Fig. 5). By further utilizing the *C. lymberakisi* assembly (subfamily Ischnocolinae I) and *P. murinus* (subfamily Harpactrinae), we were able to identify 2 candidate regions for putative DkTx-like pseudogenes in *C. lymberakisi* (Figure S10), but no functioning DkTx-like genes, in line with the distribution of DkTx homologs in Fig. 5a. In *P. murinus*, we located one candidate pseudogene spanning 59 nucleotides (Figure S10).

Phylogenetic analysis of single-domain data from the 20 identified DkTx-like sequences revealed a clear split between the N- and C-terminal domains, suggesting a single-domain duplication event, although with fairly low support (bootstrap support = 74) (Fig. 5b). The domain phylogeny also shows a deep split between the 2 single-ICK orthologs in *Acanthoscurria* from the remaining domains in DkTx, whose combined nucleotide and peptide sequence data cluster among the C-terminal DkTx-like domains (Figure S14g). The split between the monovalent *Acanthoscurria* peptides and the remaining orthologs is equally pronounced in the prepropeptide phylogeny of the DkTx homologs (bootstrap support > 95) (Fig. 5c; full-length peptide data in Figures S11b and 12b), suggesting that the domain duplication in the DkTx-like peptides occurred after the split between Theraphosinae and the 2 other subfamilies ∼110 MYA (Foley et al. 2021). The mechanism by which this duplication occurred remains unknown, however, the linker and the C-terminal tail of the second domain in DkTx show only 17% identity (2/12 identical residues). This lack of similarity indicates that domain expansion may have occurred by a different mechanism than in the Hc1a subfamily.

Analysis of the different single-domain DkTx variants, ie the 3 in Ornithoctoninae (U1-Hh1a, U1-Hh1b, and U1-Hh1c; the latter only in domain and full-sequence alignment due to lack of functioning prepropeptide), one in Poecilotheriinae (U1-Pv1a), and the U1-Ar1a- and U1-Aj1a-peptides in in *Acanthoscurria* (*A. juruenicola* and *A. rondoniae*), showed that the latter 2 peptides and U1-Hh1c closely resembled the C-terminal DkTx-domain, whereas U1-Hh1a and U1-Pv1a cluster better with the N-terminal domain (Figure S14e-h). To examine whether these monovalent DkTx homologs represent examples of domain loss or ancestral single-domain paralogs, we performed a similar search for evidence of domain pseudogenization as with the Hc1a-like peptides. Unlike the monovalent Hc1a-like peptides, the monovalent DkTx-like peptides contained neither signals of a second DkTx-like domain in the UTRs nor any resemblance of the bivalent DkTx-like UTRs in the coding domains (Figure S14e-h). However, we detected multiple TE insertions in the 3′UTRs of all monovalent peptides, some of which corresponded with TE insertions in the 3′UTRs of their closest related bivalent peptide (Figure S14e-h), suggesting that TEs could have facilitated both domain expansion as well as loss in the DkTx orthologs. Indeed, close examination of 2 contigs—one from a bivalent DkTx ortholog in *Citharognathus hosei* (Data S2: U1-Ch2a) and one from *H. hainanum* (Data S2: U1-Hh1c) containing an incomplete monovalent DkTx ortholog—revealed the presence of TE insertions immediately preceding the coding sequences of the 2 peptides. For the bivalent ortholog in *C. hosei*, which was detected in the venom (Figure S16), the entire prepropeptide has been substituted by a >1,000 bp Tc1/Mariner DNA transposon, whereas we find a Gypsy-type LTR retrotransposon immediately preceding the second domain of the monovalent *H. hainanum* ortholog, resulting in pseudogenized transcripts (Figure S14h; see Figure S17 for raw RNA read coverage spanning the transcript assemblies). For the latter monovalent peptide, the TE is not present in the otherwise identical DkTx-like paralog, suggesting domain loss and pseudogenization may be associated with retrotransposition. Taken together, domain loss has likely occurred on at least 3 occasions in the DkTx subfamily: once through loss of domain 1 and pseudogenization in *H hainanum*, and twice through loss of domain 2 in *Poecilotheria* and *Haplopelma* (Fig. 5). Although these 3 events correlate with the presence of TEs in the flanking regions of the retained domain, genomic data is needed to further test the molecular mechanisms underlying the origin of these single-domain sequences.

## Discussion

The venoms of Australian funnel-web spiders harbor diverse arsenals of ICK-type toxins (Pineda et al. 2020) and have emerged as a source for developing novel insecticides (King and Hardy 2013) and therapeutic leads (Chassagnon et al. 2017; Redd et al. 2021). However, the lack of genomic resources has hampered our understanding of the molecular mechanisms underlying the evolution of these toxin arsenals. Our results suggest that ICK toxins may have been recruited into the venom of spiders after the duplication of a nontoxin ICK gene that is still shared across Araneae, based on the exclusive expression in nonvenom gland tissues of orthologs of the 2 ICK genes identified in *R. nishihirai* and the presence of at least one of these in all genomes examined here. Although the timing of recruitment remains uncertain, the ICK toxin-encoding genes subsequently underwent a large expansion that, compared to the few available mygalomorph spider genomes, appears to be particularly large in the Atracidae family (4-fold increase compared to *M. yani*; You et al. 2024).

Although they do not account for the hundreds of toxins previously hypothesized to comprise funnel-web spider venom (Palagi et al. 2013), the expansion of ICK toxin genes reaching more than 40 paralogs is remarkable. Such a proliferation of ICK toxin genes could be facilitated by TEs showing elevated densities near ICK-encoding genes in the genome of *H. cerberea*. This suggests that TEs are associated with the expansion of ICKs, which could result in accelerated gene family expansion through the prevalence of highly similar genomic elements that increase the likelihood of NAHR. We also found a particular abundance of the Tc1/Mariner superfamily, which is a common superfamily among arthropods (Petersen et al. 2019) and is reported as a likely driver for the expansion of short, linear toxins in the venom of the Australian green-head ant (*Rhytidonopera metallica*; Isaksen et al. 2025). As class II DNA elements, Tc1/Mariner transpose by a cut-and-paste mechanism involving recognition of the terminal inverted repeats (TIR) by the transposase upon transposition (Plasterk et al. 1999). Due to the accumulation of similar or near-identical TIRs surrounding a gene in close proximity, the transposase could bind 2 TIRs on opposite sides of a gene—similar to gene capture by read through during Helitron transposition (Krasileva 2019)—effectively cutting out and pasting a gene flanked by similar TIRs. Retention of haplotypes containing both the original and new, transposed, copy would result in gene duplication through transposition, yielding a patchy distribution of closely related paralogs similar to what we observe in *H. cerberea*. However, more contiguous genomic data are required to further investigate the roles of transposition versus NAHR in the expansion of ICK paralogs, and to further evaluate the observed correlation between TEs and ICK genes in Mygalomorphae.

A particularly interesting subfamily of toxins identified in venoms of Atracidae is the bivalent Hc1a-like peptides. Our results show that these in fact represent an ancient group of bivalent toxins with an origin dating back to early in the history of mygalomorph spiders (predating the >150 MYA split between Macrothelidae and Atracidae; see Opatova et al. 2020), which suggests that its distribution is considerably wider than previously recognized (Chassagnon et al. 2017). Since then, the Hc1a subfamily has undergone dynamic evolution, including domain expansion (trivalency in U1-Pm2A), multiple domain losses (monovalency in Hc3a, U1-My1a group, U1-Hg3a-group, and most likely Hm1a-group), and a gene duplication (U21-Hc1a). Moreover, there appears to be multiple interleaving lineages with complete absence of the peptide. However, distinguishing between loss of entire orthologs, pseudogenization, divergence, lack of detection, or absence expression is difficult to evaluate without additional data. Interestingly, every instance of domain loss across the Hc1a subfamily involves a bias toward retaining the N-terminal domain. This domain in isolation retains its potent inhibition of ASIC1a (Chassagnon et al. 2017). In contrast, the C-terminal domain by itself exhibits no detectable inhibition and has instead been suggested to either potentiate the effect of Hi1a (Chassagnon et al. 2017) or produce a “pro-open” state that explains the incomplete inhibition of the channel (Berger and MacLean 2024). This functional asymmetry seems to favor preservation of the full (eg PcTx1 and Hm3a) (Escoubas et al. 2000; Er et al. 2017) or main part of the N-terminal domain (eg Hc3a) (Budusan et al. 2024) through ongoing selection. In line with experimental data (Escoubas et al. 2000; Chassagnon et al. 2017; Er et al. 2017; Budusan et al. 2024), our results emphasize the importance of the N-terminal domain in ASIC1a modulation, whereas the C-terminal domain mainly acts in augmenting receptor avidity.

Our initial hypothesis on the origin of bivalency in Hc1a was that it evolved through exon shuffling of ICK domains, which is the most common pathway for multi-domain protein repeats to evolve (Björklund et al. 2006). However, exon shuffling usually involves recombination between the vastly larger introns (Moore et al. 2008), leading to intronic separation of the resulting domain repeats. Although we found large introns separating the signal-, pro-, and mature-peptide exons in all ICK-encoding genes, the lack of introns between the 2 ICK domains of bivalent Hc1a- and U21-Hc1a peptides means exon shuffling is highly unlikely. Instead, these findings imply that bivalency evolved by local intra-exon duplication.

Local tandem duplication can occur through replication slippage through the formation of secondary structures (eg hairpins, cruciform). The hallmark of hairpin formation is the presence of palindromic repeats, where the stability of hairpin formation is proportional with the length of the inverted repeating unit (Viguera 2001; Looman et al. 2002; Björklund et al. 2006). However, we did not detect inverted repeats within the different domains, rendering slippage an unlikely scenario for Hc1a evolution. Another potential mechanism involves merging and translocation of 2 independent single-ICK toxins by retrotransposition, which is the proposed mechanism for the evolution of the bivalent CpTx1 in the araneomorph spider *Cheiracanthium punctorium* (Vassilevski et al. 2010). However, the lack of a single-ICK Hc1a precursor gene and identical retrotransposons in the immediate vicinity of the third exon refutes also this hypothesis.

Instead, the high interdomain similarity, nonintronic separation of domains, and high similarity between interdomain linker and C-terminal tail, indicate bivalency in the Hc1a subfamily likely evolved through NAHR within the third exon region—a mechanism that also likely underlies both domain expansion (U1-Pm2a) and at least one case of domain loss (Hc3a). Intra-exon duplication also led to an exaptation of the N-terminal tail into a linker domain, which is supported by the high similarity between the linker and C-terminal tail. The interdomain linker probably plays a crucial role in orienting the 2 domains for optimal functionality (eg Ramanujam et al. 2024) and has remained highly conserved over evolutionary time.

Like Hc1a, bivalency appears to also have evolved only once in the DkTx subfamily, although the domain split of DkTx orthologs shows lower bootstrap support than the split between Hc1a domains. However, contrasting the early evolution of bivalency in Hc1a, we found that bivalency in DkTx-like peptides stems from a more recent evolutionary event and is probably restricted to just 2 subfamilies within the Theraphosidae. In addition, both the N-terminal domain and interdomain linker show low similarity to the C-terminal domain and tail, respectively. This suggests that bivalency in DkTx has evolved through a different genomic mechanism than in the Hc1a subfamily, such as by transposition or NAHR between the third exons of single-domain ICK paralogs. The presence of multiple TEs in the 3′UTRs of single-domain DkTx peptides is consistent with this representing a potential mechanism. In particular, we identified a likely recent domain loss and pseudogenization of a paralog otherwise identical to a bivalent DkTx orthologue in *H. hainanum* that was coexpressed with an LTR retrotransposon. Although genomic resources are required to test this hypothesis, our observations suggest ongoing modification in the DkTx-like toxins that correlate with TE presence.

Although bivalency is a recurrent feature of evolutionary innovation across ICK toxins (Bohlen et al. 2010; Vassilevski et al. 2010; Chassagnon et al. 2017; Maxwell et al. 2018; Liu et al. 2023; Maxwell et al. 2023), we find that bivalency has evolved on just a single occasion in each of the 2 toxin families examined here. These findings are consistent with previous studies implicating that convergent recruitment of architectural changes—not including reversal to monovalency—is rare (Gough 2005). They also suggest that the Hc1a and DkTx families do not necessarily share structural or functional characteristics that make them more prone to repeatedly evolve bivalency. Instead, mechanisms of genetic rearrangement, such as transposition and NAHR, could also be facilitated by the numerous, closely related paralogs that comprise ICK toxins, thereby increasing the probability of evolving bivalency. These findings, and the widespread distribution of the Hc1a subfamily, highlight ICK toxins from spiders as a promising source of novel, bivalent toxins.

## Materials and methods

### DNA extraction and sequencing

We extracted genomic DNA from whole-body tissue of 1 female individual of *H. cerberea* using a combination of the QIAGEN Genomic-Tip 100/G and QIAGEN MagAttract*®* HMW DNA extraction kit for animal tissue. The specimen had been preserved in 100% ethanol until DNA extraction. High-molecular-weight DNA (33 µg) was size-selected with short read elimination (>10 kb) prior to library preparation with the PacBio protocol for Preparing HiFi Libraries SMRTbell® ExpressTemplate Prep Kit 3.0. The resulting library was sequenced in 2 rounds a PacBio Revio 25 M SMRT Cell by the Norwegian Sequencing Centre, yielding a total of 49.92 Gb across 4,082,190 HiFi reads (mean read length ∼ 12.2 kb) (Table S1d).

### Genome assembly and annotation

We used the HiFi reads to assemble the genome of *H. cerberea* using hifiasm v0.15.1 (Cheng et al. 2021) with default settings, following adapter removal with TrimGalore (Martin 2011; Krueger et al. 2023). We used Jellyfish v2.3.0 (Marçais and Kingsford 2011) to perform a k-mer count (*k* = 21 and *k* = 32) of the HiFi reads, and plotted the k-mer frequency distribution to estimate genome size and zygosity (Figure S1b). Quality assessment of the primary assembly was first performed with gfastats v1.3.6 (Formenti et al. 2022). Second, we applied Merqury v1.0 (Rhie et al. 2020) to perform reference-free quality control and completeness assessment of the primary assembly using k-mers (*k* = 21) (Figure S1c). We then evaluated assembly completeness with the BUSCO software v5.4.3 (Manni et al. 2021) using the arachnida_odb10 dataset, and used BlobToolKit v.4.3.11 (Challis et al. 2025) to visualize the contiguity, GC content and completeness of the assembly with a snailplot (Figure S1a). Due to the moderate coverage (13X), large assembly size (7.8 Gb), and no strong duplication signals in the assembly (Figure S1c), we did not attempt to purge potential duplicate haplotigs for risk of over purging. Putative contaminant sequences were detected using FCS-GX v0.4.0-3-g8096f62 (part of NCBI's Foreign Contamination Screen) (Astashyn et al. 2024) and removed (126 contigs) from the final assembly. Lastly, we used RagTag v2.1.0 (Alonge et al. 2022) to scaffold the nuclear genome assembly against the near-chromosome level reference assembly of *M. yani* (GCA_039090855.1). The output was only used to investigate the chromosomal distribution of Hi1a-like genes and was not included in the final draft assembly.

To annotate protein-coding genes in the resulting assembly, we first softmasked the genome for repetitive DNA (see *Repeat Annotation*), and used BRAKER3 (Gabriel et al. 2024) for de novo gene prediction. We ran BRAKER3 on 64 threads with default settings, using a database consisting of (i) gene sets from *Parasteatoda tepidariorum* (GCA_000365465.3), *Stegodyphus dumicola* (GCA_010614865.2), and *Stegodyphus mimosarum* (GCA_000611955.2) in combination with the (ii) single-copy protein sequences from the arachnida_odb10 BUSCO database and (iii) transcriptomic data from venom glands and whole body tissue of *H. cerberea* (same individual used for genome assembly; see *Transcriptome Assembly*). Gene set completeness was evaluated with BUSCO v5.4.3 (Manni et al. 2021) using the arachnida_odb10 dataset (Table S1b).

Aiming to obtain a curated set of ICK-encoding genes in *H. cerberea*, we first extracted putative ICK entries from the peptide sequences predicted by BRAKER3 by manual investigation of all predicted protein entries based on conserved cysteine frameworks and suggested classifications from Pineda et al. (2020). Second, we used blastp (BLAST v.2.14.1) to search for more ICKs among the predicted proteins using our extracted ICK sequences as query. Third, we evaluated the genes encoding the resulting ICK peptide dataset by manual investigation of gene annotations in the *H. cerberea* assembly using the integrated genome viewer (IGV v.2.12.3; Robinson et al. 2011). Gene boundaries were curated using evidence from RNA datasets from *H. cerberea* (venom glands and whole-body tissue) that were mapped to the genome assembly using HiSAT2 v.2.2.1 (Kim et al. 2019) and publicly available translated transcriptomes from 2 *H. cerberea* and *Hadronyche formidabilis* individuals (Accession ID: PRJEB14734), which were mapped using miniprot v.0.13 (Li, 2023).

To remove potentially unresolved allelic ICK variants that are not true paralogs in the assembly, we dot-plotted the contigs harboring ICK genes against each other using minimap v.2.29 (k = 19) (Li, 2018). The output was visualized with D-GENIES (Cabanettes and Klopp 2018, Figure S18), where the shorter of 2 completely overlapping contigs were removed.

The ICK genes on the remaining contigs were clustered using CD-HIT v.4.81 (Fu et al. 2012) using a 95% sequence identity threshold for clustering (amino acid, full gene and coding sequence, see Table S7). For each cluster, we looked at the read depth along the contigs by mapping raw PacBio HiFi reads with minimap v.2.29 (Li, 2018). In combination with dot-plots of the respective contigs of a cluster using MMseqs2 search (*k* = 15, minimum sequence identity >20%) (Steinegger and Söding 2017) and visualization of ICK gene coordinates with pyGenomeTracks (Lopez-Delisle et al. 2021), we evaluated whether an ICK gene overlapped with another ICK gene in a region of low read depth. The genes were retained if they displayed unique flanking regions and read depth above the 13X average (Figure S19).

Putative function of the curated ICK genes was predicted by sequence homology and structural similarity to known sequences using FoldSeek (van Kempen et al. 2024) and the nonredundant protein database from the BLAST server. Putative cleavage sites for signal peptides were predicted using SignalP v.5.0 (Almagro Armenteros et al. 2019). Finally, the ICK gene set (mature-, prepro- and full-peptide sequences) was clustered using CLANS (amino acid sequence) (Frickey and Lupas 2004) and MMseqs2 (nucleotide sequence) (Steinegger and Söding 2017) to infer gene families (Figure S2).

### Repeat annotation

To obtain a species-specific TE library for *H. cerberea*, we used RepeatModeler2 v.2.0.1 (Flynn et al. 2020) for de novo prediction of TEs using the *H. cerberea* genome assembly as input. We included the LTR structural detection module (Ellinghaus et al. 2008; Ou et al. 2018) to better identify LTRs in the genome. This output a raw TE library, which was processed with TEtrimmer (Qian et al. 2025) for automatic curation. In brief, TEtrimmer applies a BLAST-extend-clustering workflow of each TE consensus predicted by RepeatModeler2, which is an iterative search, alignment, clustering, and extension of similar sequences to obtain full-length TE consensus sequences with correctly defined boundaries. TEtrimmer also uses CD-HIT-EST (Fu et al. 2012) to remove duplicated sequences, discards low-copy TEs with no typical terminal motifs as false positives, and classifies the output library. We ran TEtrimmer with default settings, which yielded a nonredundant automatically curated TE library. This library was further processed by applying a similar strategy as described in Aasegg Araya et al. (2025), where we first used the nonredundant output library to mask the *H. cerberea* assembly using RepeatMasker v.4.1.5 (Smit et al. 2013) to obtain a priority list for curation, prioritizing insertions in (i) the vicinity or overlapping Hc1a-like genes, (ii) insertions on contigs with ICK-encoding genes, and (iii) overall genomic abundance. Second, we classified the consensus sequences with PASTEC (Hoede et al. 2014), and performed manual curation of a selection of consensus sequences according to our priority list (Table S8). Manual curation was performed using structural and homology-based evidence TE-Aid (Goubert et al. 2022), which is included in the TEtrimmer output, and from PASTEC. For incomplete sequences, we applied a manual BLAST-extension-alignment protocol to identify the true boundaries of the consensus sequences (eg identification of target site duplications). Chimeric sequences were partitioned, curated and reclassified using the Dfam (Storer et al. 2021) and Censor (Kohany et al. 2006) databases. Finally, this subset of manually curated TE consensus sequences was used to mask and automatically curate the restoring library for 3 consecutive rounds. This resulted in a species-specific, manually curated TE library for *H. cerberea* with an increase in long, classified consensus sequences compared to the raw RepeatModeler2 output (Table S4 and Figure S20). We used the resulting TE library to mask the *H. cerberea* assembly with RepeatMasker v.4.1.5 using the slow search setting for high sensitivity searches. This library, in combination with Censor (Kohany et al. 2006) was also used to mask Hc1a- and DkTx-like transcripts.

### Repeat analysis of ICK-encoding genes

To evaluate the association between TEs and genes encoding peptides of the ICK fold, we first extracted the 36 contigs harboring ICK-encoding genes and visually compared their repeat landscape (ie frequency of TE insertions against the Kimura divergence to their respective consensus) to the repeat landscape of the total 6,771 contigs with gene annotations. We then extracted the flanking regions (+/− 5,000 bp) of each annotated gene (genes that did not have flanking regions of that size in both ends were excluded for comparability) and calculated the density of (i) all TEs, (ii) only DNA elements, and (iii) only retroelements within each flanking region. The resulting densities were compared between flanking regions of ICK genes versus flanking regions of the same number of non-ICK genes (selected randomly and repeated 1,000 times). Next, we focused on the 2 contigs harboring the Hc1a subfamily (contigs 1,124 and 6,199) to investigate the TE insertions surrounding Hc1a, U21-Hc1a, and Hc3a. First, we plotted the TE density (all TEs, only DNA elements, and only retroelements) within nonoverlapping sliding windows of 100 bp for the gene regions including flanking regions of 5,000 bp. Density estimates were visually compared with the mean TE densities on the respective contigs. Lastly, in order to compare the composition of TE insertions between the 3 genes, we filtered the annotated TEs based on pairwise shared TE insertions (at the level of TE families) and used geneviewer v.0.1.10 (van der Velden 2025) for visualization of syntenic TE insertions at the level of TE families.

### Comparative genomic analyses

We downloaded 7 long read-based genome assemblies from Mygalomorphae scaffolded to near-chromosome level (see Table S1c). We evaluated the completeness of these assemblies using BUSCO v5.4.3 (Manni et al. 2021) and provide a comparison of other available assembly metrics including the *H. cerberea* assembly in Table S1c. To search for ICK paralogs corresponding to the ICK gene set in *H. cerberea*, we used miniprot v.0.13 (Li, 2023) to map our 42 predicted ICK-encoding genes onto the 7 mygalomorph assemblies. Each hit was inspected manually using IGV v.2.12.3 (Robinson et al. 2011) for validation and filtered on full-length hits with identified start and stop codons. For the 2 assemblies included from Theraphosidae *C. lymberakisi* (accession ID: GCA_964291345.1), *P. murinus* (accession ID: GCA_053477515.1), and Euctenizidae (*A. stephencolberti*, accession ID: GCA_051312355.1) (Table S1c), we also searched for conserved motifs of DkTx and Hc1a using nucleotide HMMs (Eddy 2011) of the ICK domains in Hc1a and DkTx (see *Sequence Analysis of Hc1a- and DkTx-like Peptides* for HMM construction).

### Venom collection

The female individual of *H. cerberea* was collected in 2020 from the Central Coast, NSW, Australia with the assistance of the Australian Reptile Park (Gosford, NSW, Australia) and housed individually at 23-25 °C in a plastic container. Forceps were used to aggravate the spider until venom was secreted and collected continuously by aspiration until we could obtain no more venom. The venom was lyophilized and stored at −30 °C prior to shipment.

In addition, a total of 11 spider species of Mygalomorphae (see full species list in Table S5) were purchased from Exo-Pet (https://www.exo-pet.de) and Zoohaus W&S (https://www.zoohaus-ws.de) and housed individually at 23 °C in plastic containers. Of these, 10 were milked exhaustively for venom (only adult or subadult specimens) through biting over a plastic film and electrically stimulating the venom glands for secretion until we could not obtain more venom. The venom samples were immediately dissolved in 5-10 µL MilliQ water (depending on the amount of obtained venom) and snap frozen on liquid nitrogen.

### Venom profiling

A total of 11 venom samples (3-5 µL) were either dissolved in 80 mM ammonium bicarbonate (samples that were stored in MilliQ water) or resuspended in 10% acetonitrile (ACN) in 100 mM ammonium bicarbonate (lyophilized venom) and subsequently reduced with DTT and alkylated with iodoacetamide. The reduced and alkylated samples were digested by incubating with a concentration of ∼20 ng/mL trypsin overnight at 37 °C. The digested samples were washed and resuspended in a final concentration of 5% ACN and 1% formic acid, prior to peptide identification by LC-MS/MS on a UHPLC-TimsTOF Pro mass spectrometer (Bruker). The MS/MS spectra were searched against the transcriptomic sequence databases custom to each sample using MaxQuant (Cox and Mann 2008) in DDA mode, using a default decoy-based false discovery rate of 0.01%. Carbamidomethylated cystines was set as a fixed modification, C-terminal amidation was set as a variable modification, and up to 2 missed cleavages were permitted. Due to low identification numbers for some samples, we also repeated each search with MSFragger (Kong et al. 2017) using the same search parameters as above. The output for each search was evaluated and processed with an in-house filtering script that uses SignalP v.5.0 (Almagro Armenteros et al. 2019) to predict signal peptides and sort the database of confidently identified sequences.

### Transcriptome assembly

We downloaded every publicly available venom gland transcriptomic dataset from the order Aranea and whole-body transcriptomic data from Mygalomorphae (total of 156 datasets from 72 unique species, see data availability for Mygalomorphae in Figure S7) from the Sequence Read Archive (SRA; last accessed August 20, 2023) and added 3 venom gland transcriptomes (3 unique species) from previously collected data (see full species list in Table S5). We only included datasets that were sequenced with paired-end sequencing. To obtain better phylogenetic coverage of relevant tissue (venom glands or whole-body including venom glands) from mygalomorph species, we included transcriptomes from the 11 mygalomorphs (Table S5) described in *Venom Collection.* These 11 specimens were anesthetized, and their venom glands were dissected and snap frozen in liquid nitrogen. Dissection was performed 3 days after milking. Additionally, we obtained tissue from the chelicerae, legs, prosoma, opisthosoma and venom glands from *H. cerberea* 3 days after milking, which was stored in RNAlater. Total RNA was isolated using the TRIzol® protocol (Life Technologies), except for the venom glands of *H. cerberea*, which were isolated using QIAGEN spin column purification. The quality and concentration of RNA was evaluated using a Bioanalyzer 2100 (Agilent Technologies) and NanoDrop (Thermo Fisher Scientific), and 1 µg of mRNA was used for each of 16 tissue samples to prepare the cDNA libraries with TruSeq mRNA prep. The resulting libraries were sequenced on a 1/8 NovaSeq × 25B flow cell, yielding a range of 67,497,124-230,183,650 raw fragments for the 16 sequenced samples.

The total 175 mRNA datasets from 86 unique species were trimmed using Trim Galore v.0.6.10 (Krueger et al. 2023) on paired-end mode, using a PHRED-score cutoff of 25 and minimum read length of 75 bp. Following trimming and quality control, the remaining 184 mRNA datasets were assembled with Trinity v.2.15.1 (Grabherr et al. 2011) and translated with TransDecoder v.5.7.0 (Haas 2023) using a lower limit of 50 amino acids for ORF prediction to include a broad range of peptide candidates. This resulted in a database of predicted peptides from 86 species.

### ICK expression profiles in *H. cerberea*

To evaluate the function of the different ICK-encoding genes in *H. cerberea*, we compared expression profiles of the annotated genes across the venom glands, chelicerae, prosoma, opisthosoma, and legs from the same *H. cerberea* individual. The processed RNA fragments from each tissue were mapped onto the assembly using with HiSAT2 v.2.2.1 (Kim et al. 2019) expression was counted with featureCounts (Liao et al. 2014). The raw read counts were normalized to log2-transformed counts per million (log-CPM) using edgeR (Chen et al. 2025) and then compared across tissues using pheatmap v.1.0.13 (Kolde 2025), applying Ward's minimum variance clustering (ward.D2) to group the genes, visualized as a dendrogram.

### Identification and phylogenetic inference of Hc1a-like and DkTx-like peptides

To identify peptides of putative homology to Hc1a, we searched for similar sequences within our newly generated Aranea transcriptome database using BLASTP v.2.14.1, and in online repositories using BLASTP (nonredundant and UniProtKB databases) and TBLASTN (transcriptome shotgun assembly database), using Hc1a (translated from the *H. cerberea* assembly) as query with a lenient e-value of 10. The output was combined and filtered for sequences with at least 40% sequence identity (above the “twilight zone”), followed by filtering of peptides using regular expressions (based on Hc1a-like sequence patterns: CX_2-7_C_3-10_CCX_1-4_CX_3-13_C) and manually assessed the output for putative ICK homologs. This approach was repeated 3 times with the curated output-sequences as query, in order to arrive at a manually curated set of Hc1a-homologs containing both monovalent and multivalent sequences. We used CD-HIT v.4.8.1 (Fu et al. 2012) to remove identical, redundant sequences from within each species dataset.

To analyze the evolutionary relationship between the identified Hc1a-like peptides, we aligned (i) full-peptide sequences, (ii) mature peptides, (iii) prepropeptides, and (iv) single domains, using MAFFT local pairwise alignment method (L-INS-i) with a low gap opening penalty (1.0) and including the –leavegappyregion option. Alignments were investigated manually to ensure that the cysteine frameworks were conserved. Furthermore, we aligned the nucleotide sequences of the same entries (excluding protein sequences from UniProtKB with no nucleotide sequences) using the coding sequences and proceeding 3′UTRs. For the output alignments, we used IQTREE2 (Kalyaanamoorthy et al. 2017; Minh et al. 2020) to generate ML phylogenetic trees, using default settings and ultrafast bootstrap (1,000 rounds) (Hoang et al. 2018). Due to the variability in mature peptide sequences and the high interdomain similarities of multivalent Hc1a-like peptides, we used the prepropeptide-phylogeny to make evolutionary inferences of the peptide sequences, applying information from the single-domain phylogeny, analyses of 3′UTRs (see *Structural Analysis of Hc1a-like genes*) and parsimony-principles of least number of changes to arrive at the final tree topology. Finally, we applied the same methodologies as with Hc1a to infer the evolution of DkTx (*C. schmidti*), only excluding analyses of nucleotide sequences, as we did not have genomic data to rationalize the use of coding sequences and UTRs to infer trajectories of the homologous sequences.

### Sequence analysis of Hc1a- and DkTx-like peptides

In order to assess potential molecular mechanisms involved in the evolution of bivalency in Hc1a, as well as the rapid retractions and expansion of domains in its putative homologous peptides, we applied different methods involving both genomic and transcriptomic data. First, to infer the relationship between Hc1a and its closely related monovalent Hc3a variant, we aligned the 2 sequences using the mature peptides and the nucleotide coding sequences, showing that the first and second half of Hc3a display near-identical sequence identity to different domains of Hc1a, as suggested in Budusan et al., (2024). We then aligned the 2 different domains of Hc1a (C-terminal to N-terminal domain, using peptide and nucleotide sequences), to identify potential regions of high-identity overlaps that could facilitate unequal crossing-over leading to monovalency in Hc3a, using ggmsa v.1.0.3 (Zhou et al. 2025) to display the alignments. Second, to rule out the possibility of gene rearrangement due to the formation of secondary structures, eg hairpins, cruciform, G-quadruplexes, which are known to cause both gross and local rearrangements (Wang and Vasquez 2023), we self-dot-plotted the coding sequence of Hc1a (third exon encoding the mature peptides) (Figure S15), which can reveal internal repetitive patterns such as tandem repeats and palindromes, associated with forming secondary structures. Third, to analyze the reversal of the different groups of monovalent Hc1a-homologs, we analyzed the coding sequence (CDS) of the mature peptide-encoding region and 3′UTRs of every entry that had available, full-length nucleotide sequences. Given a scenario of domain loss by frameshifts or point mutations leading to stop codons, we expected to observe remnants of the lost domain(s) in the 3′UTRs of the nucleotide sequences. In the case of domain reversal by unequal crossing-over—such as in Hc3a—we expected to observe stronger similarities between the 3′UTRs of Hc1a and Hc3a. To perform a broad search of Hc1a-like ICK variants, we extracted the single-domain sequences (nucleotide and peptide) of every identified Hc1a-homolog and created 1 nucleotide HMM profile and 1 peptide HMM profile Hc1a-like ICKs using HMMER v.3.4. The resulting HMMs were used to search for similarities in the Hc1a-like nucleotide sequences (CDS and 3′UTR) and translated sequences using every possible reading frame. In addition, we performed nucleotide sequence alignments of the representatives from the different monovalent groups against its closest bivalent ancestor, using coding sequences and 3′UTRs, and plotted sequence similarities within nonoverlapping sliding windows of 5 bp (Figure S14). Finally, we annotated putative TE insertions in the transcripts using our species-specific TE library for *H. cerberea* and the Censor TE-database (Kohany et al. 2006). These analyses were repeated for the identified DkTx homologous peptides.

To investigate the DkTx paralogs with preceding TE insertions, ie U1-Hh1c and U1-Ch2a, we mapped their raw RNAseq reads onto their respective transcriptome assemblies using HiSAT2 (Kim et al. 2019), and visualized mapping coverage with pyGenomeTracks (Lopez-Delisle et al. 2021).

## Supplementary Material

msag076_Supplementary_Data

## Data Availability

Whole genome shotgun assembly, transcriptome assemblies, and raw data have been deposited to NCBI under bioproject PRJNA1353152. Gene annotation, TE annotation, and proteomic result summaries are available via figshare (“Ancient origin and dynamic evolution of bivalent spider toxins”: https://figshare.com/s/f84d2e6f3928b922cb84). Proteomic data have been deposited to the PRIDE database under accession PXD070138.
