# Supplementary material for: Ancient origin and dynamic evolution of bivalent spider toxins
Source: Mol Biol Evol. 2026 Mar 24;43(5):msag076. doi: 10.1093/molbev/msag076 (PMC13172734; doi:10.1093/molbev/msag076)
Supplement: msag076_Supplementary_Data [file msag076_supplementary_data.zip › revision_Supplementary_Figure_Araya_et_al_Ancient_Origin_of_bivalent_toxin.pdf]

Supplementary Figures

Ancient origin and dynamic evolution of bivalent spider toxins

Robin A. Araya[1], Marius F. Maurstad[1], David Wilson[2], Lachlan D Rash[3], Mehdi Mobli[4], Kjetill S. Jakobsen[1] and Eivind A. B. Undheim[1*]

[1]Centre for Ecological and Evolutionary Synthesis, Department of Biosciences, University of Oslo, 0316 Oslo, Norway.

[2]Advanced Analytical Centre, James Cook University, Cairns, Australia

[3]School of Biomedical Sciences, The University of Queensland, St Lucia, QLD, 4072, Australia.

[4]Centre for Advanced Imaging, The University of Queensland, St Lucia, QLD 4072, Australia

* Email address for correspondence: e.a.b.undheim@ibv.uio.no

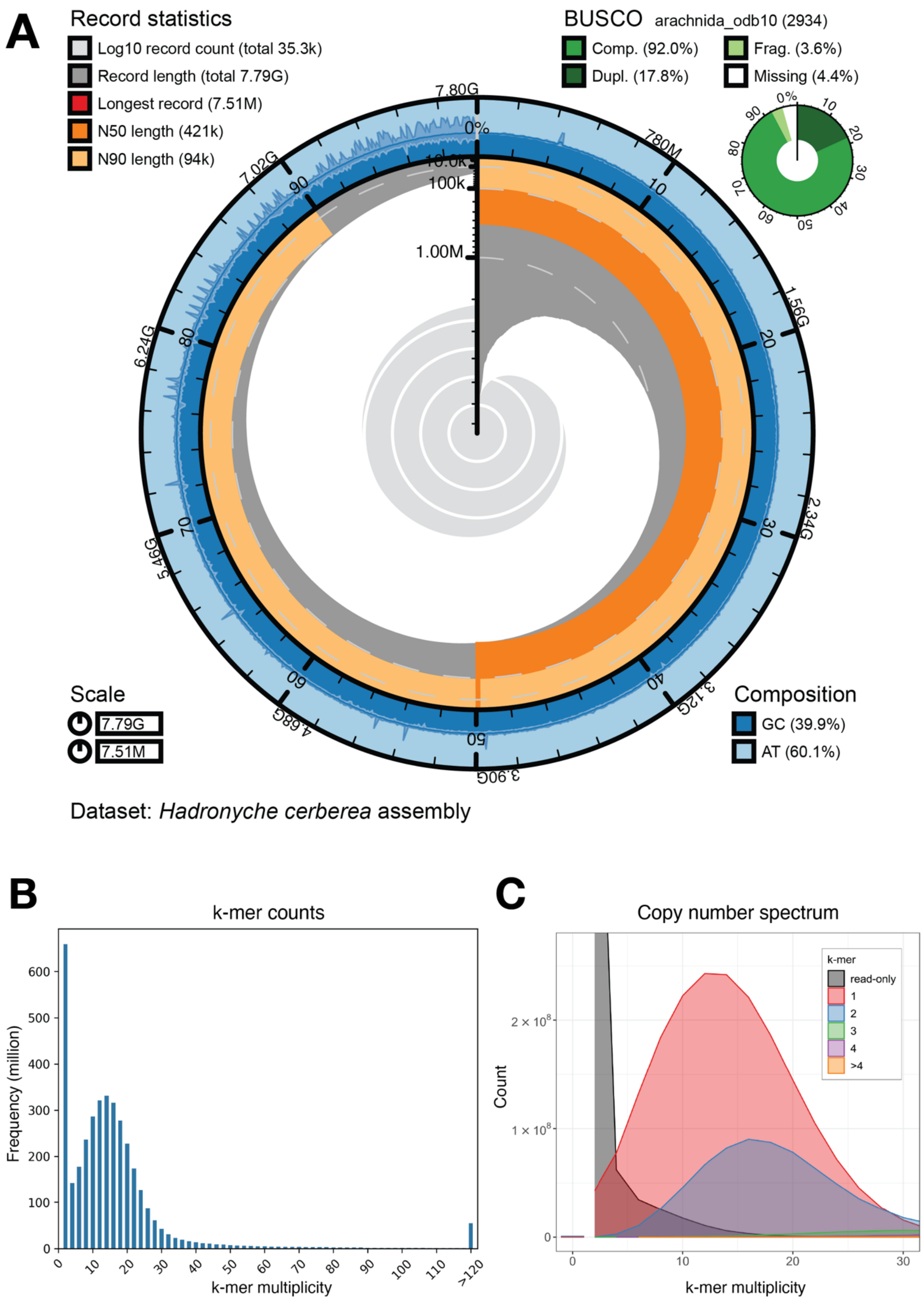

SI Figure S1: Assembly statistics for the *H. cerberea* primary genome assembly. A) Snail-plot summarizing the length distribution and nucleotide composition of contigs, including assembly metrics and BUSCO completeness using the arachnida_odb10 database. **B)** K-mer distribution (k=21) for all HiFi-reads from *H. cerberea*. **C** Copy number spectrum of the same k-mers as in **B**, coloured according to the legend.

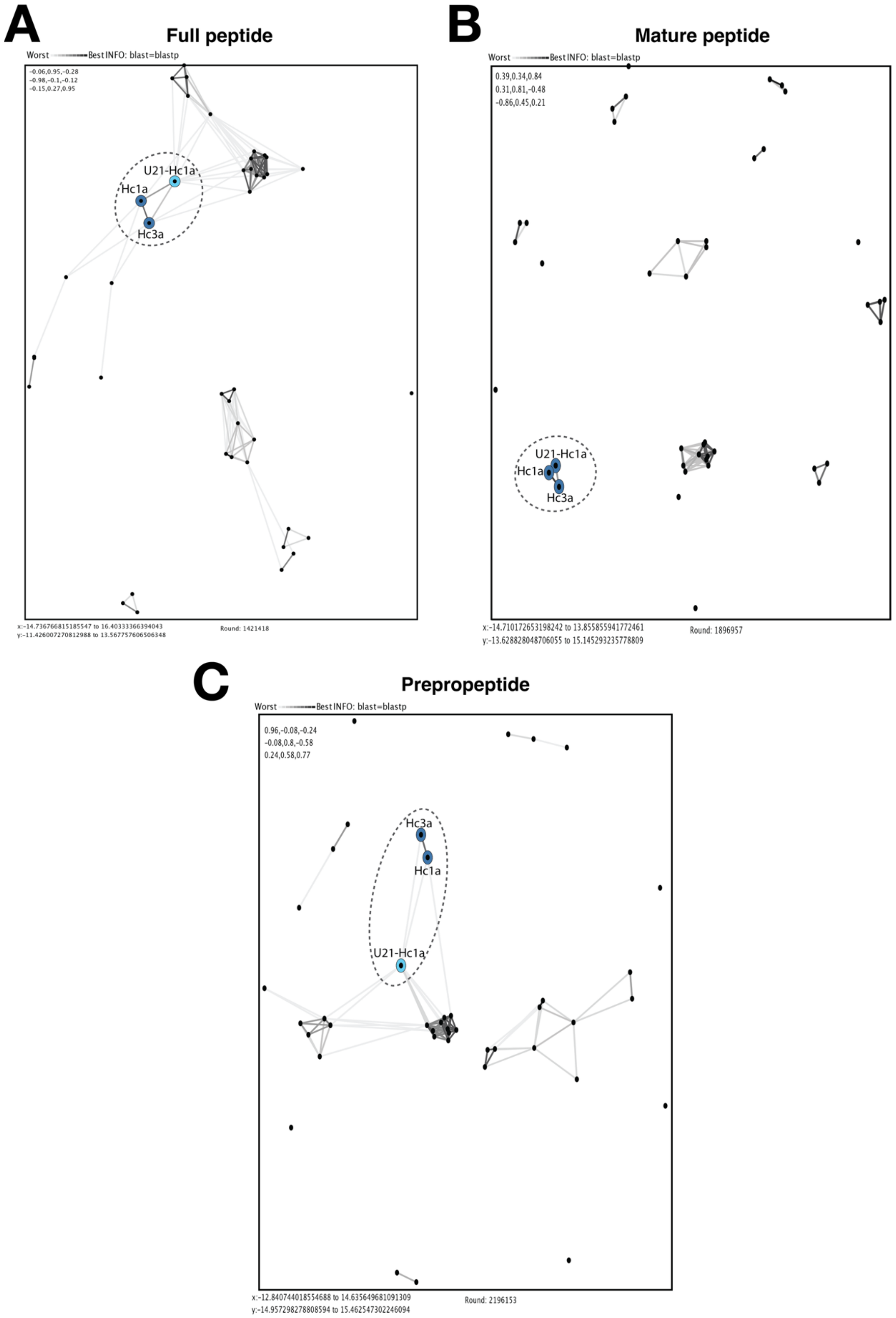

SI Figure S2: Gene clustering of ICK peptides in *H. cerberea*. Pairwise blastp of the translated peptides from 42 ICK-encoding genes from *H. cerberea* are clustered with CLANS. Black dots are peptide sequences, and the Hc1a gene cluster is highlighted within dotted circles and coloured according to the clustering of the corresponding coding sequences for **A)** full peptide, **B)** mature peptide and **C)** prepropeptide.

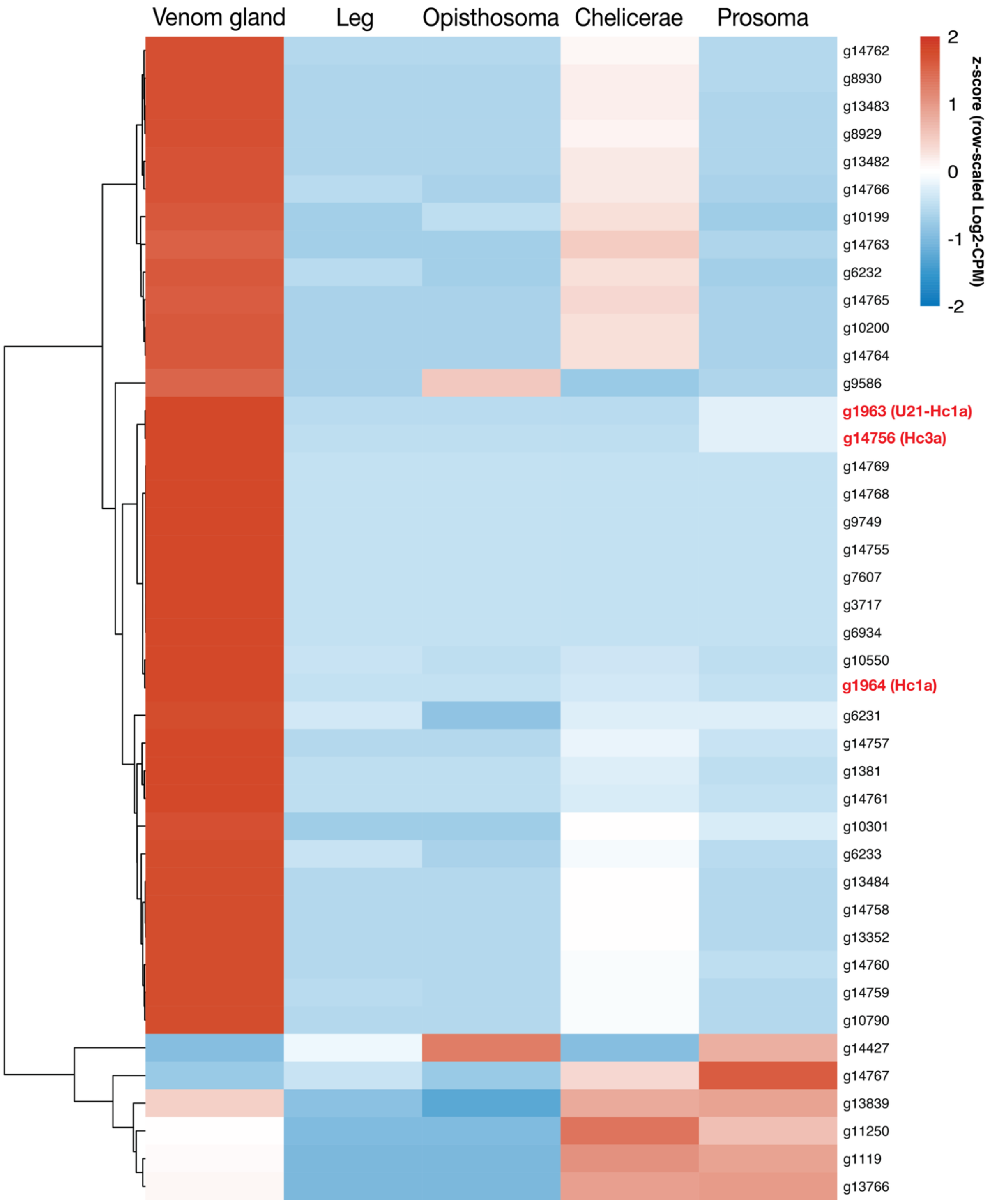

SI Figure S3: Expression profiles of ICK-encoding genes in *H. cerberea*. Heatmap showing RNA expression of ICK genes in *H. cerberea* across different tissues. Values are given as row-scaled log2-CPM (counts per million), where dark red is relative high expression and dark blue is low expression as indicated in the upper-right scale bar. Gene identifiers are shown on the right, corresponding to the braker IDs in SI Table S2, and members of the Hc1a gene subfamily are highlighted in bold red.

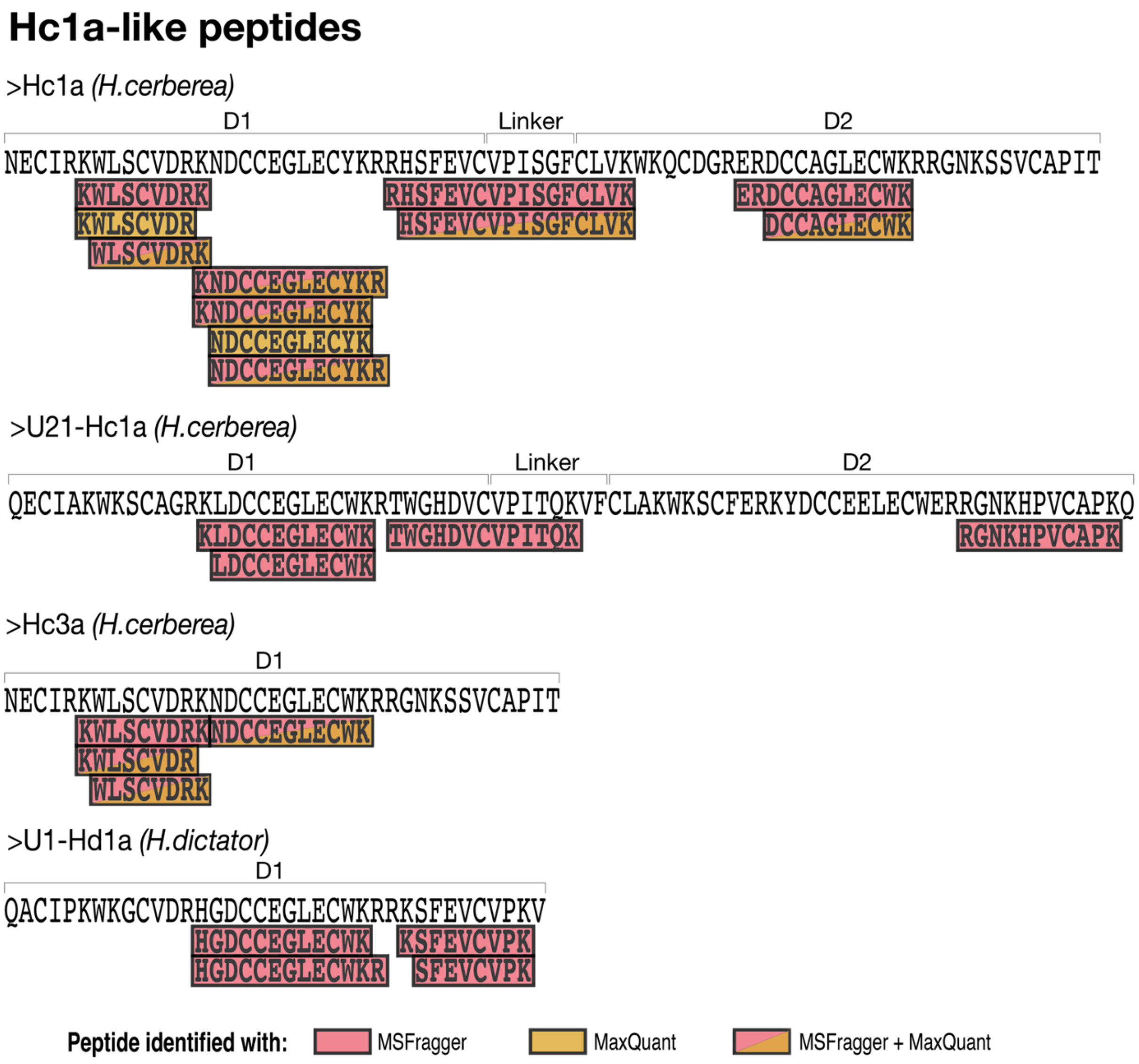

SI Figure S4: Mass spectrometry-identification of Hc1a-like peptides. Alignment of tryptic peptide fragments to Hc1a orthologs across Mygalomorphae. Peptides are coloured according to the software that was used to identify peptide spectrum matches (red: MSFragger only, yellow: MaxQuant only, red and yellow: identification by both software).

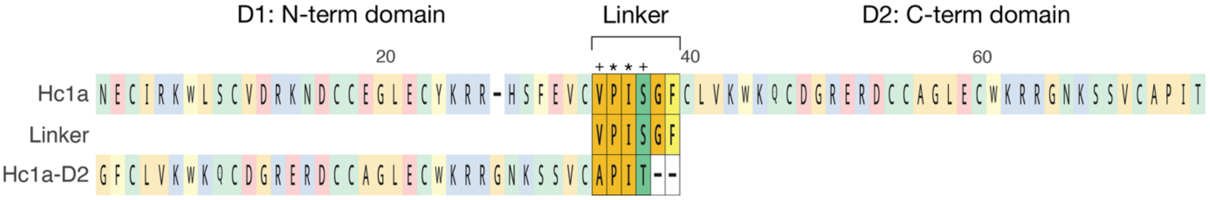

SI Figure S5: Sequence similarity between linker and tail of C-term domain in Hc1a. Sequence alignment of the full mature peptide (top), linker (middle) and C-terminal domain (bottom) of Hc1a. Identical residues are marked with an asterisk (*), similar residues (same properties) are marked with a plus sign (+), gaps are shown as dashes (-). Residues are coloured according to physicochemical properties. The linker residues are highlighted in bright colours, whereas domain residues are shown with lower opacity.

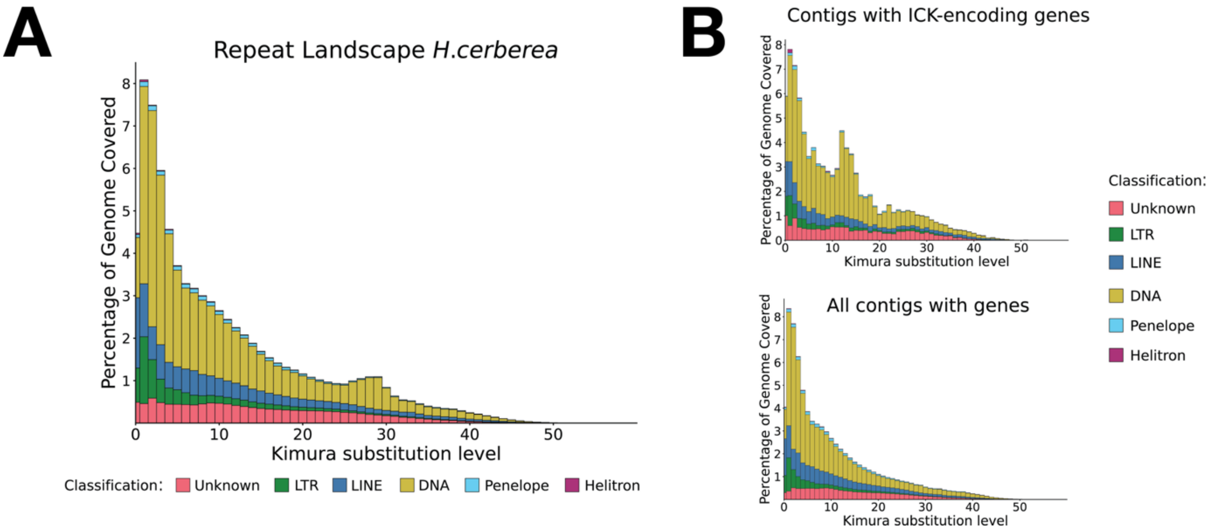

SI Figure S6: Comparison of ICK- and non-ICK contigs. Repeat landscape for only the contigs harbouring ICK-encoding genes (top) and all contigs harbouring genes (bottom), showing the genomic coverage of TEs (%) plotted against its divergence from their respective consensus sequences (Kimura substitution level) on the x-axis. TE orders are coloured according to legend.

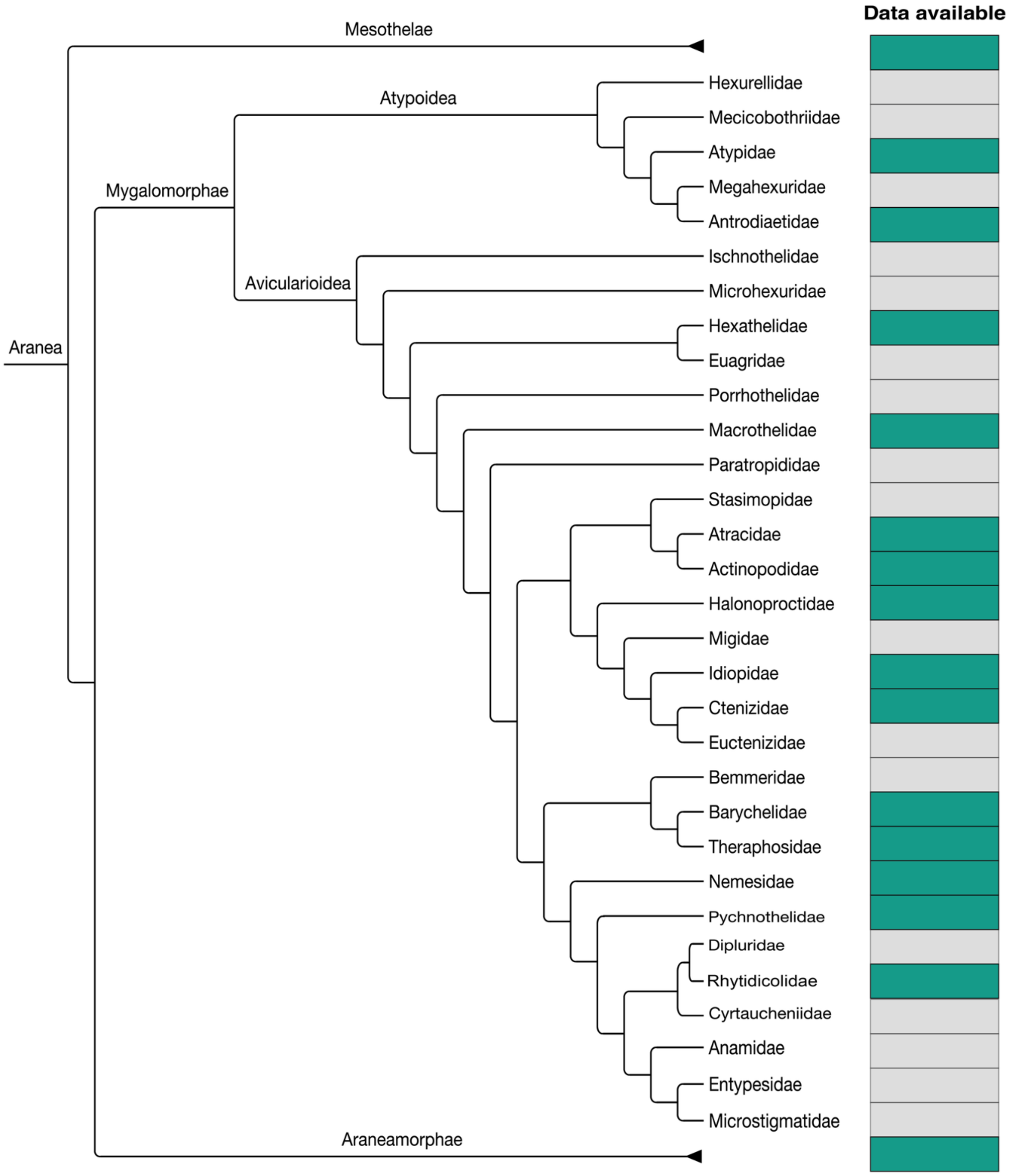

SI Figure S7: Available transcriptomic data from Aranea, highlighting Mygalomorphae. Overview of available transcriptomic data from venom glands and/or whole body in Mygalomorphae, Mesothelae and Araneamorphae. Families with available data used in this study (SI Table S5, accessed from SRA on August 20, 2023) are coloured green, families with no available data are coloured grey.

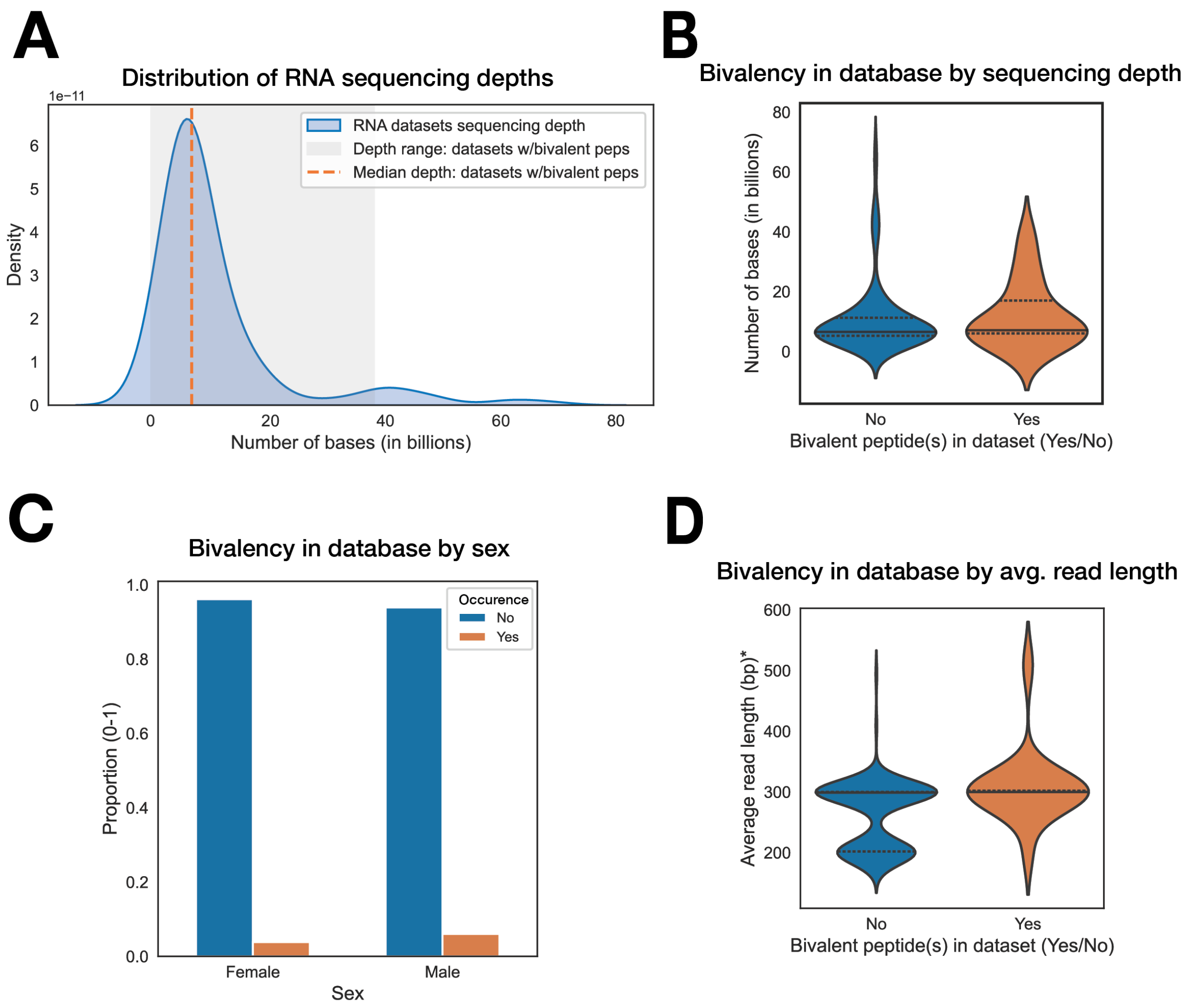

SI Figure S8: Descriptive statistics of spider RNA database. A) Kernel density estimate-plot showing the distribution of RNA sequencing depths in the individual transcriptome datasets used in our Aranae database, showing number of bases (in millions) plotted against the relative frequency (y-axis) in the database. Median depth is shown as a stapled red line, and depth range of RNA datasets with identified bivalent peptides is illustrated by the grey shaded area. **B)** Violin plot showing the distribution of sequencing depths (number of bases in millions) across datasets with (orange) and without (blue) identified bivalent peptides. **C)** Proportion of datasets with (orange) and without (blue) identified bivalent peptides according to sex. **D)** Violin plot showing the distribution of average read lengths across datasets with (orange) or without (blue) identified bivalent peptides.

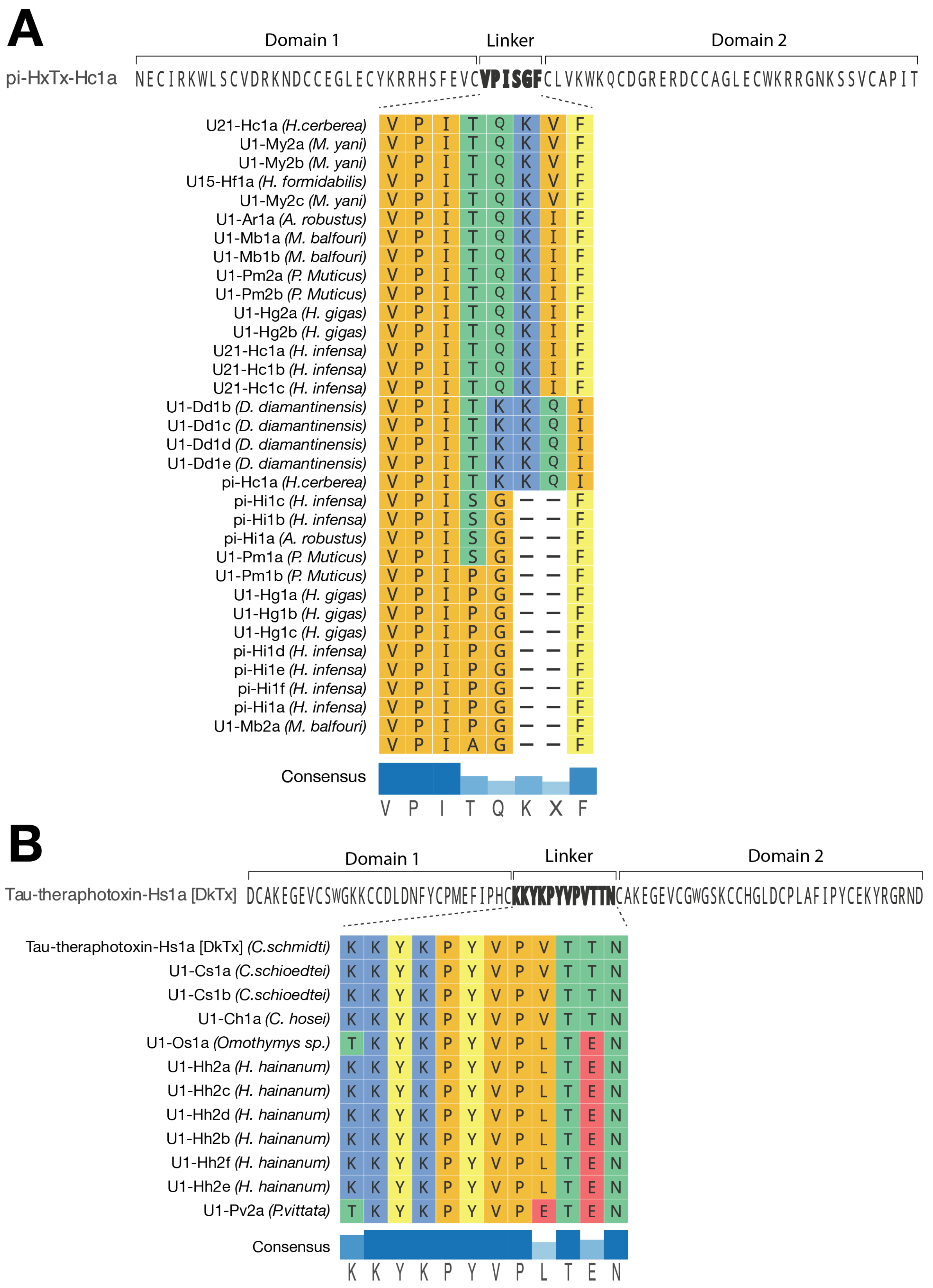

SI Figure S9: Linker alignments of multivalent toxins. Alignments of the peptide linkers from multivalent **A)** Hc1a orthologs and **B)** DkTx orthologs. Annotated mature peptides of the reference sequence (**A**: Hc1a, **B**: DkTx) are shown above the aligned linker peptides, coloured according to physicochemical properties. Consensus linker sequences are displayed below each alignment.

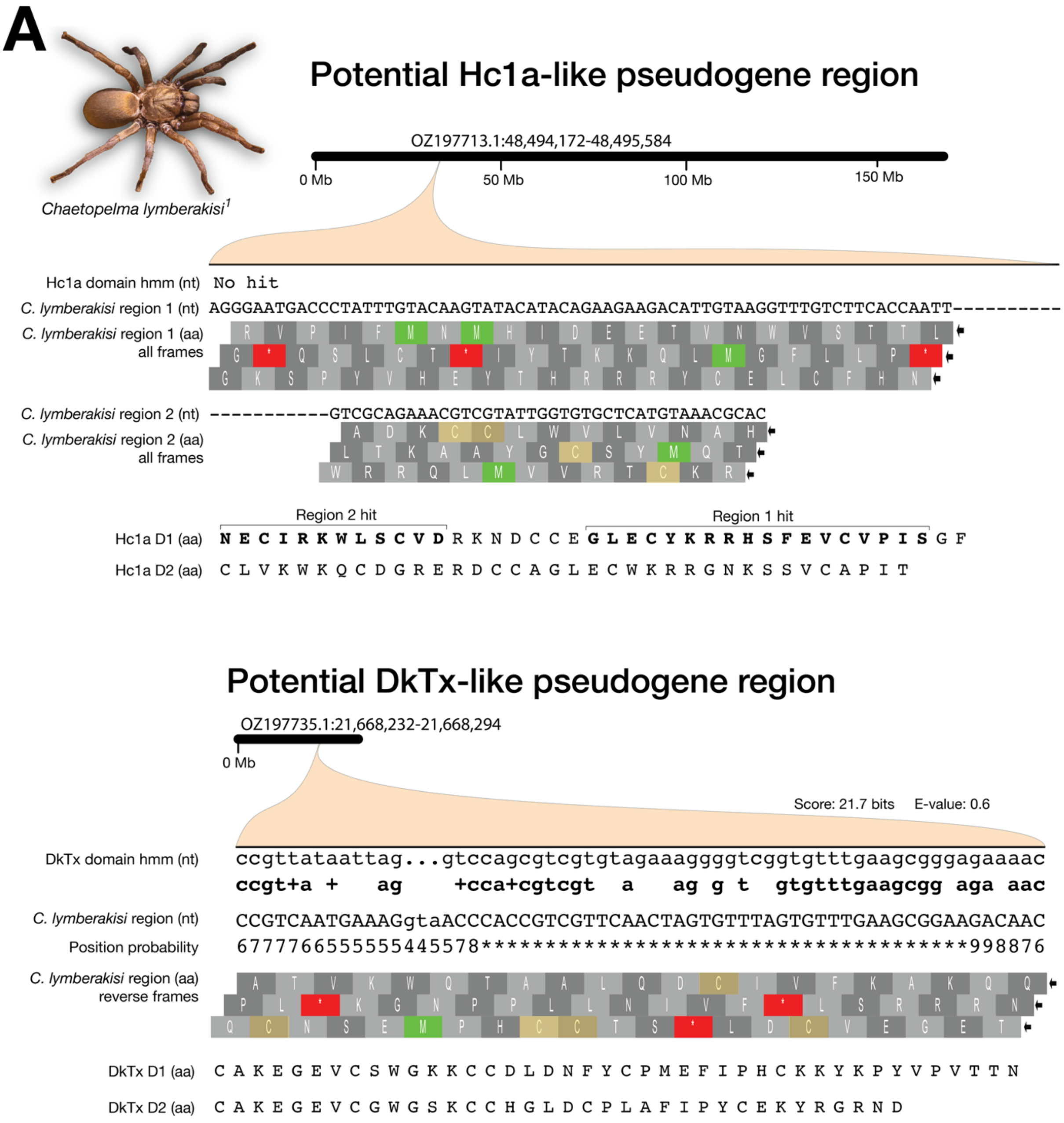

SI Figure S10: Putative Hc1a- and DkTx-pseudogene regions in *Chaetopelma lymberakisi* and *Pterinochilus murinus*. Scaffolds harbouring putative pseudogenes are shown as black, horizontal lines with positions shown in megabases (Mb) and pseudogene coordinates displayed above each scaffold, highlighted in beige. **A)** Potential pseudogene regions for *C. lymberakisi* for Hc1a (top) and DkTx (bottom), with corresponding output from HMMER (identical nucleotides between nHMM and pseudogene region shown in bold, position probability from 1: low probability to *: high probability), translations in three frames displayed below the nucleotide sequence and ancestral peptides (Hc1a and DkTx) shown at the bottom, partitioned by domains (plus linker or tail). If there are hits to regions of the ancestral peptide with miniprot, it is shown in bold. [1]Image by Nils Heller.

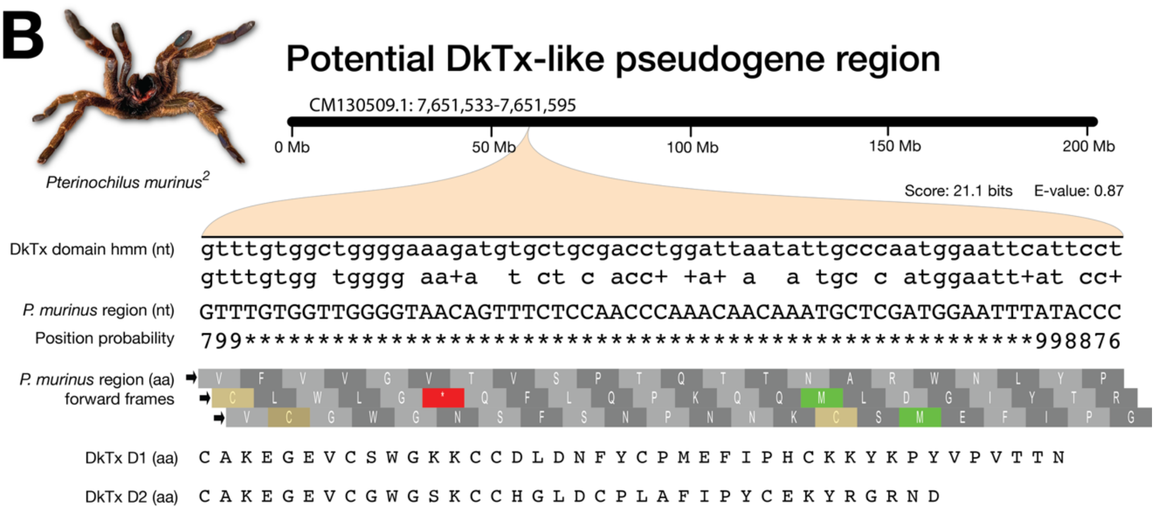

SI Figure S10 (continued): Putative Hc1a- and DkTx-pseudogene regions in *Chaetopelma lymberakisi* and *Pterinochilus murinus.* Scaffolds harbouring putative pseudogenes are shown as black, horizontal lines with positions shown in megabases (Mb) and pseudogene coordinates displayed above each scaffold, highlighted in beige. **B)** Potential pseudogene regions for *P. murinus* for DkTx, with corresponding output from HMMER (identical nucleotides between nHMM and pseudogene region shown in bold, position probability from 1: low probability to *: high probability), translations in three frames displayed below the nucleotide sequence and ancestral peptides (Hc1a and DkTx) shown at the bottom, partitioned by domains (plus linker or tail). If there are hits to regions of the ancestral peptide with miniprot, it is shown in bold. [1]Image by Rainer Peter Yusuf Vierkoetter.

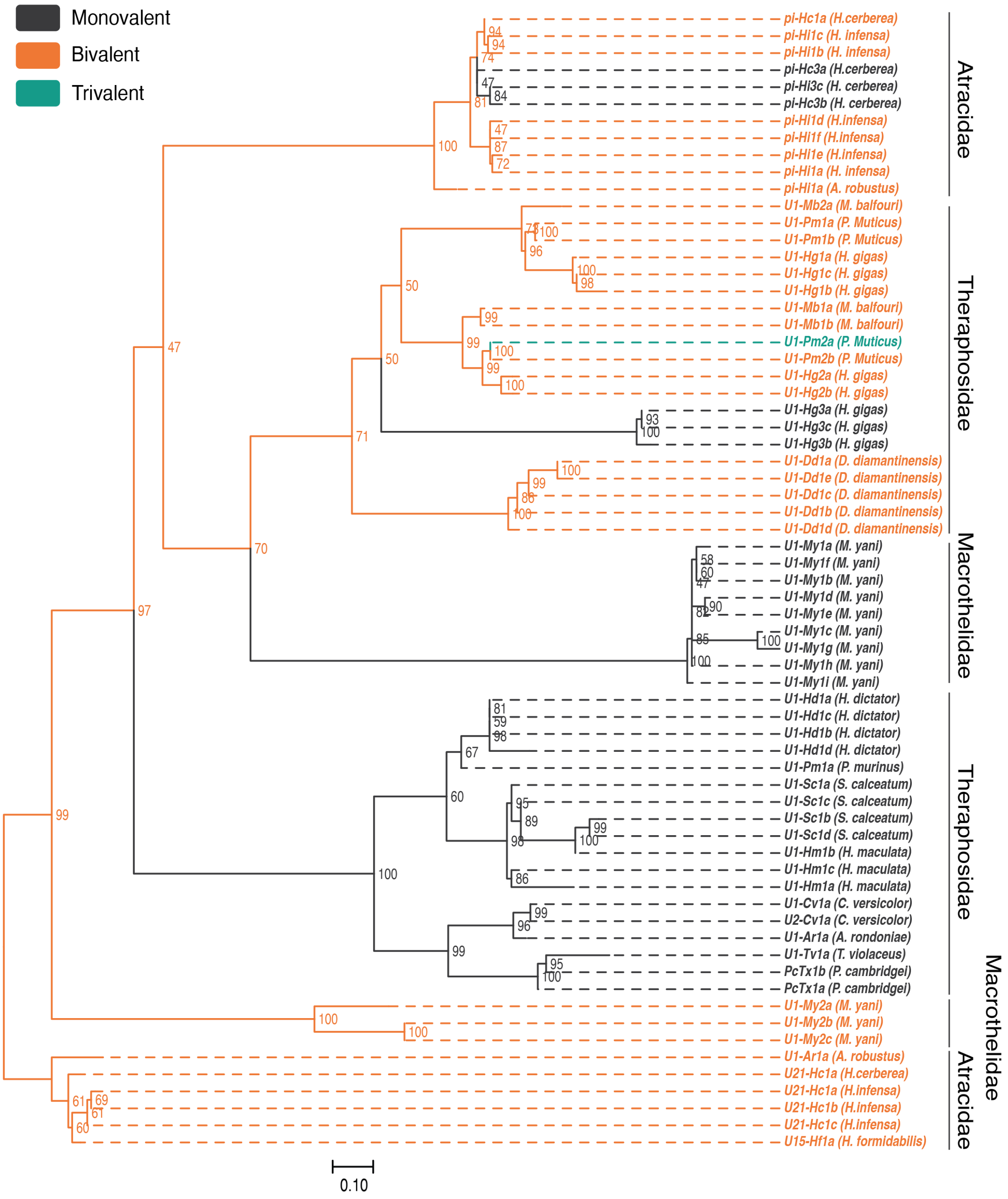

SI Figure S11: ML phylogeny of full-length bivalent orthologous peptides. A) Hc1a full-length orthologous peptide sequences. The phylogeny was rooted against the U21-Hc1a group (bottom clade). Toxin names correspond to IDs in **SI Table S6,** and the family is shown on the far-right side. Bootstrap values are shown as numbers, and branches with bootstrap support <70 are collapsed. Branch and name colour are according to legend, corresponding to mono-, bi-, or trivalency.

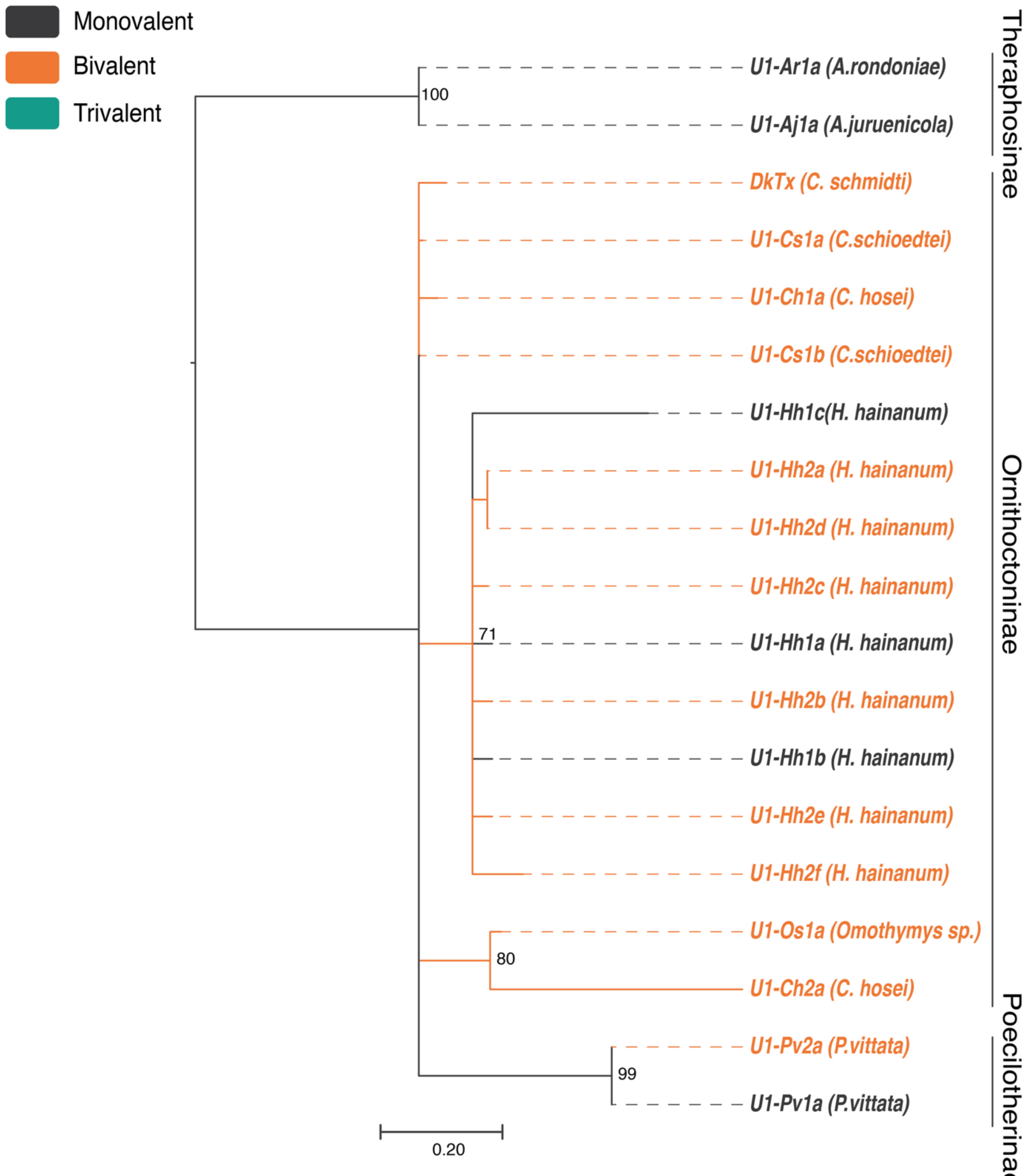

SI Figure S11 (continued): ML phylogeny of full-length bivalent orthologous peptides. B) DkTx full-length orthologous peptide sequences. The phylogeny was rooted against the Theraphosinae-group (top clade). Toxin names correspond to IDs in SI Table S6, and the family is shown on the far-right side. Bootstrap values are shown as numbers, and branches with bootstrap support <70 are collapsed. Branch and name colour are according to legend, corresponding to mono-, bi-, or trivalency.

Hc1a orthologs

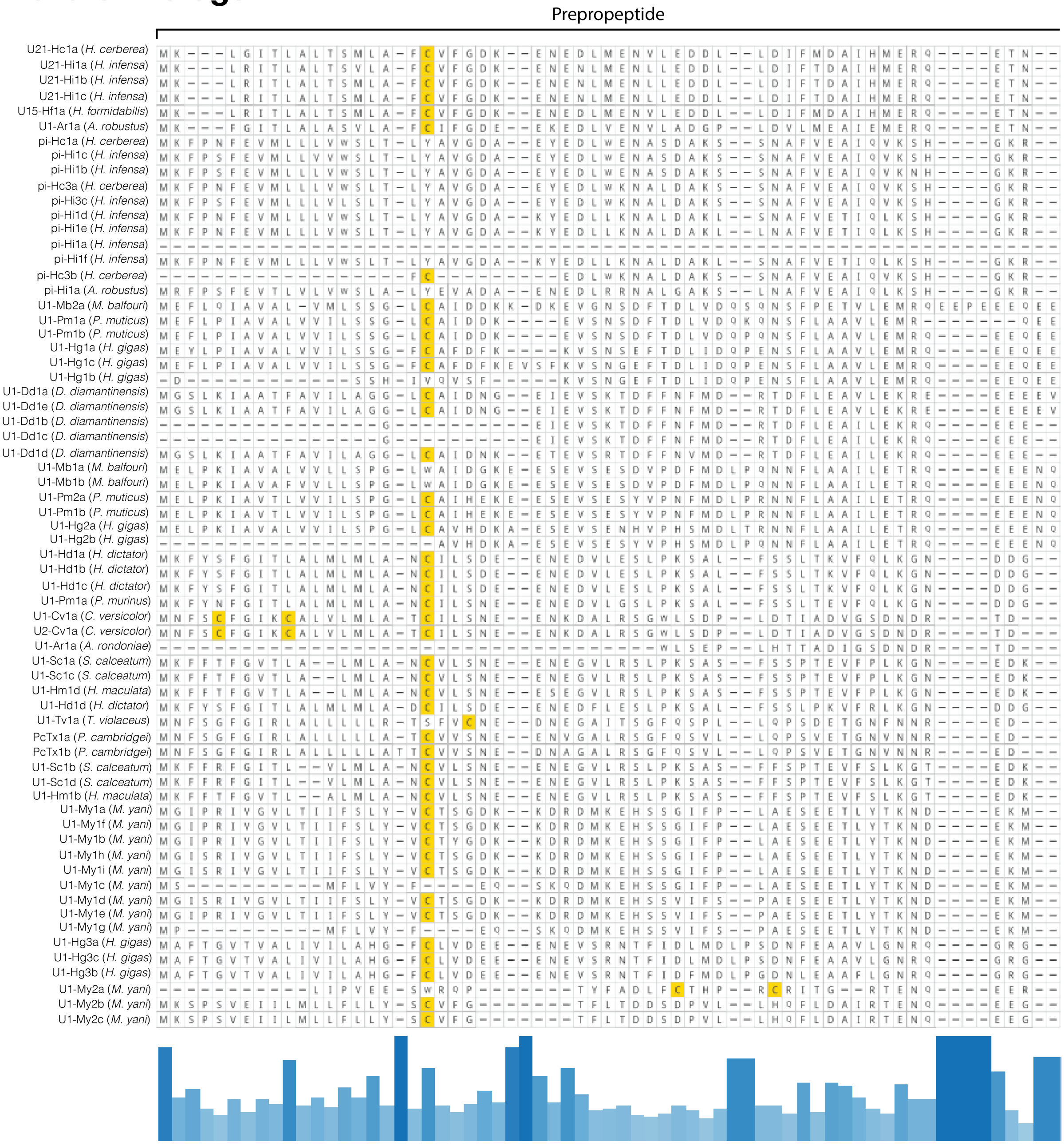

SI Figure S12: Sequence alignments of bivalent orthologs. A) Multiple sequence alignment of Hc1a-like orthologous peptides. Cysteine residues are highlighted in yellow and region annotation is displayed on top of the alignment. Sequence conservation is shown as blue bars under the alignments, where light blue indicates low conservation and dark blue indicates high conservation.

Hc1a orthologs

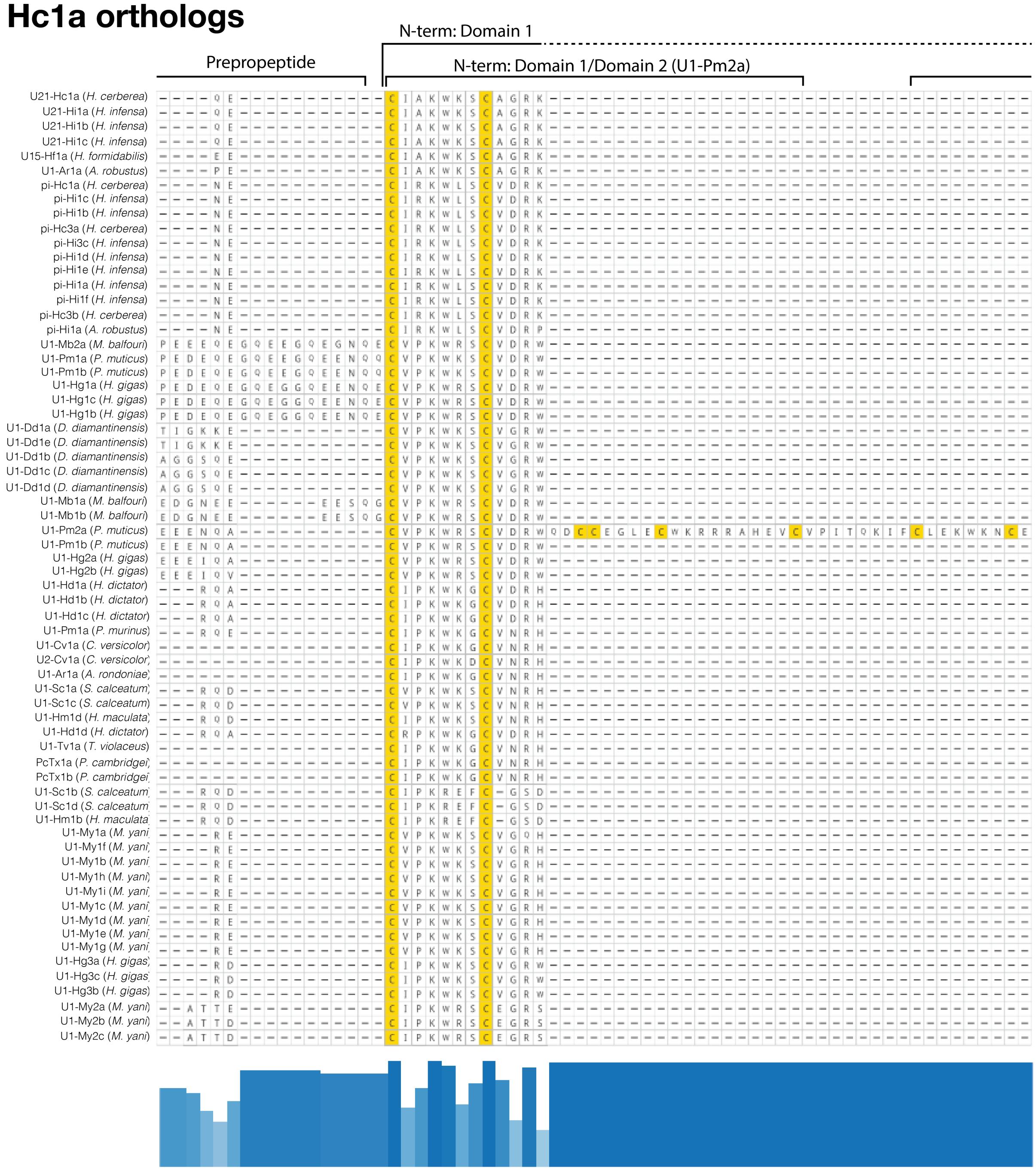

SI Figure S12 (continued) : Sequence alignments of bivalent orthologs. A) Multiple sequence alignment of Hc1a-like orthologous peptides. Cysteine residues are highlighted in yellow and region annotation is displayed on top of the alignment. Sequence conservation is shown as blue bars under the alignments, where light blue indicates low conservation and dark blue indicates high conservation.

Hc1a orthologs

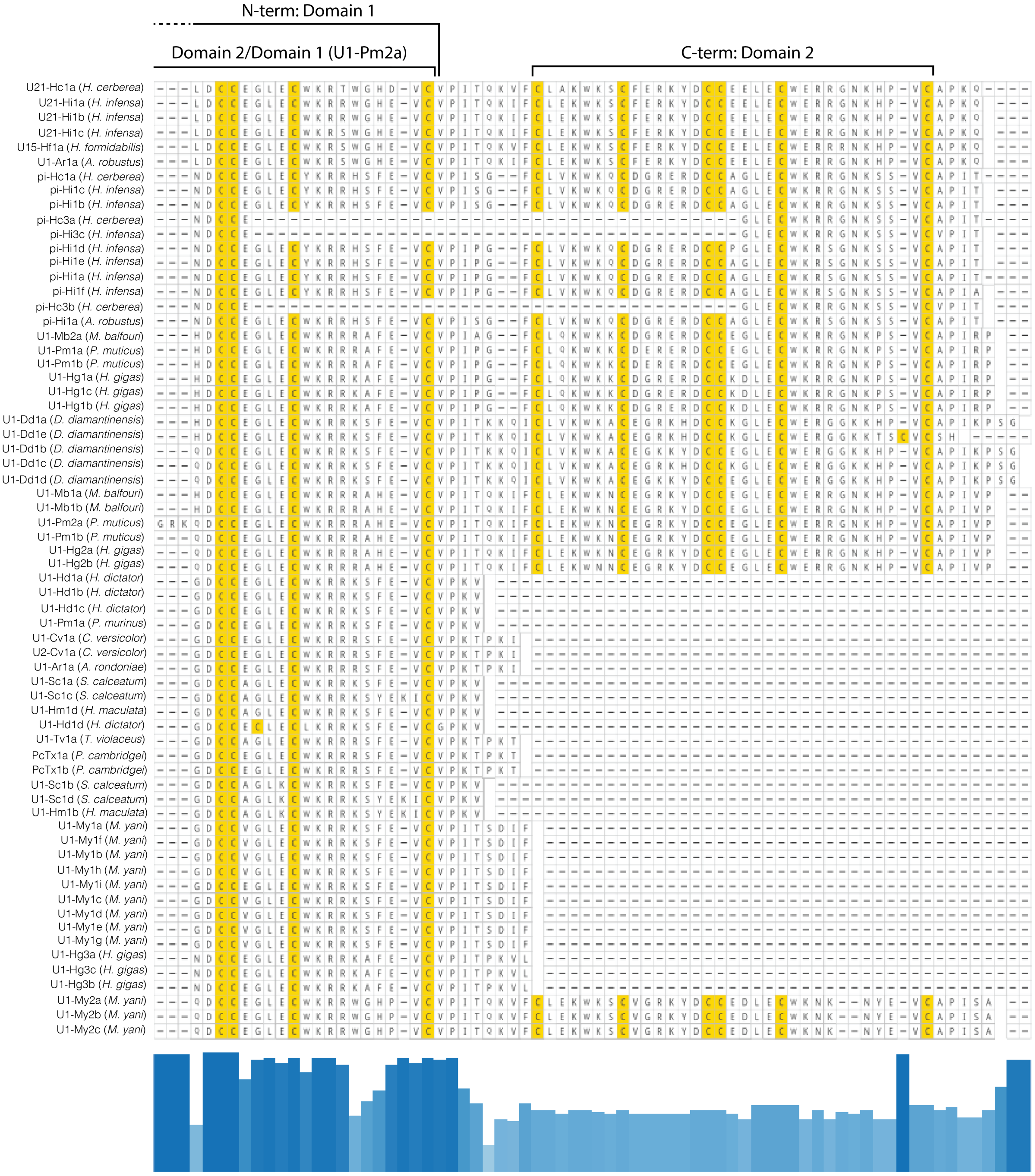

SI Figure S12 (continued) : Sequence alignments of bivalent orthologs. A) Multiple sequence alignment of Hc1a-like orthologous peptides. Cysteine residues are highlighted in yellow and region annotation is displayed on top of the alignment. Sequence conservation is shown as blue bars under the alignments, where light blue indicates low conservation and dark blue indicates high conservation.

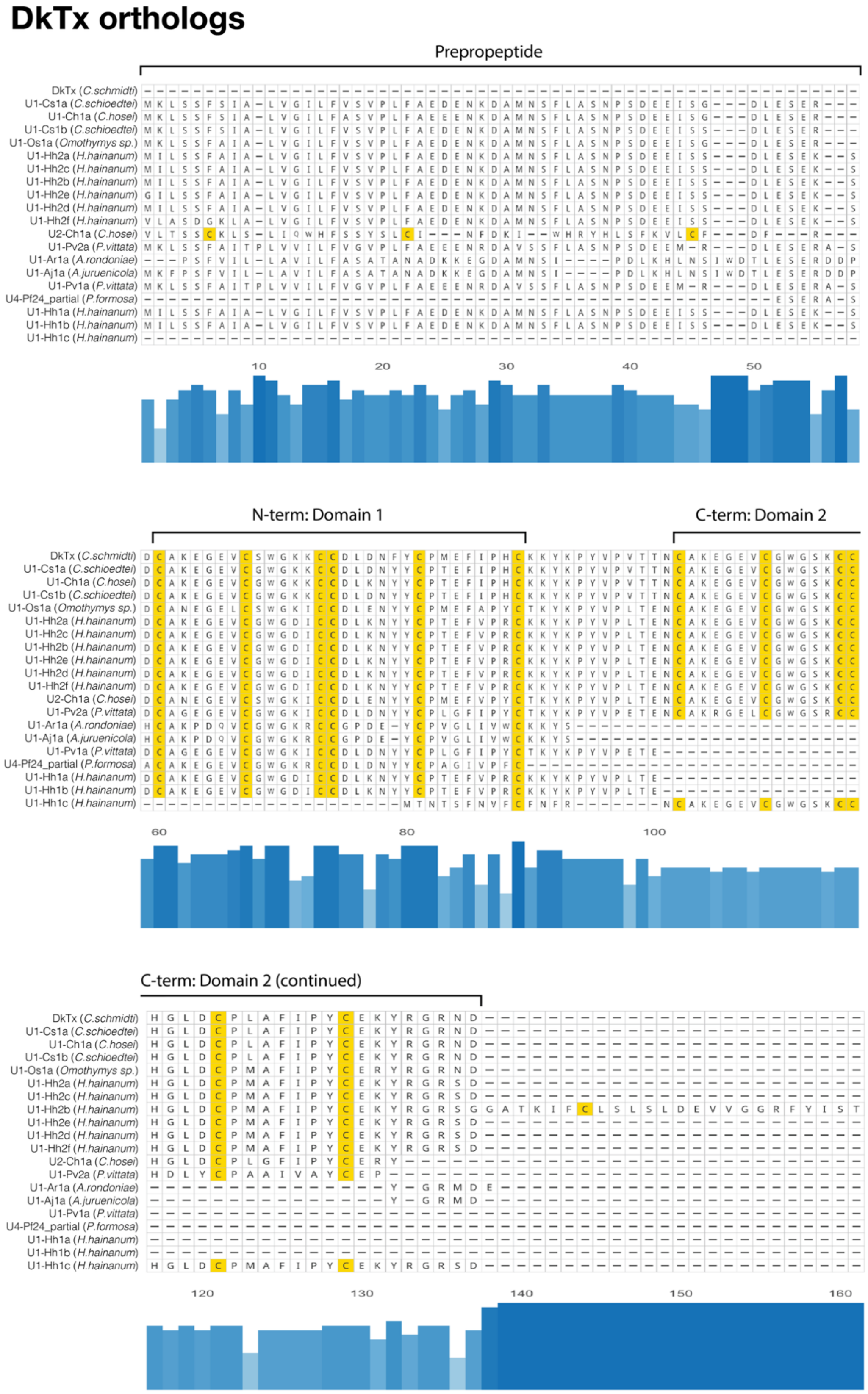

SI Figure S12 (continued): Sequence alignments of bivalent orthologs. B) Multiple sequence alignment of DkTx-like orthologous peptides. Cysteine residues are highlighted in yellow and region annotation is displayed on top of the alignment. Sequence conservation is shown as blue bars under the alignments, where light blue indicates low conservation and dark blue indicates high conservation.

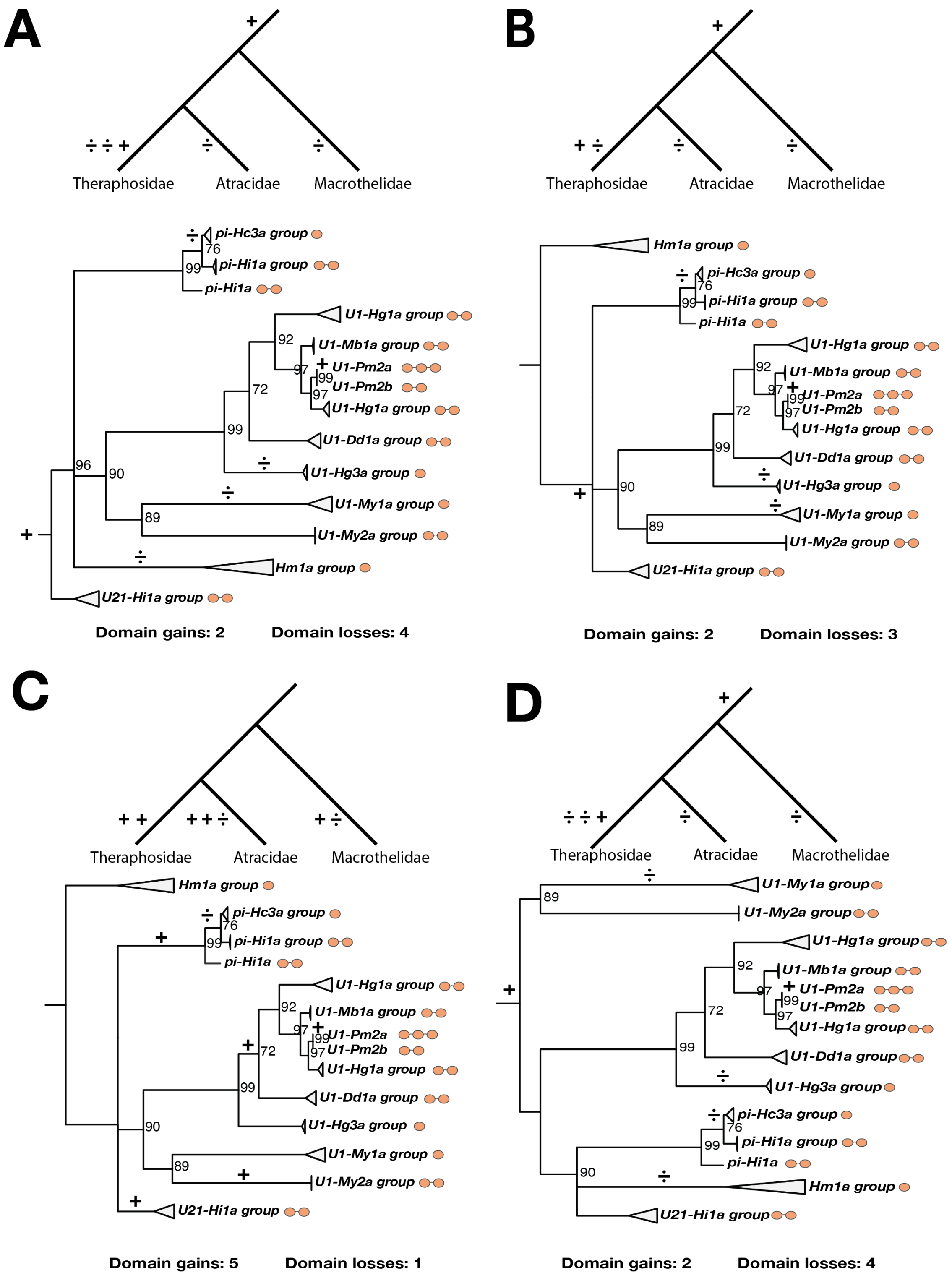

SI Figure S13: Alternative tree topologies of Hc1a orthologous peptides. Domain gain (+) and loss (-) on the top family tree are annotated on the corresponding peptide phylogeny, based on different rooting of the ML prepropeptide phylogeny in Figure 3C. The number of domain gains and losses are displayed under each topology, whereas the topology in **A** displays the topology used in **Figure 3C.** Similar peptide groups have been collapsed (grey triangles) and their Hc1a configuration (mono-, bi- or trivalent) is shown as orange circles. Bootstrap values are shown as numbers, and branches with bootstrap support <70 are collapsed.

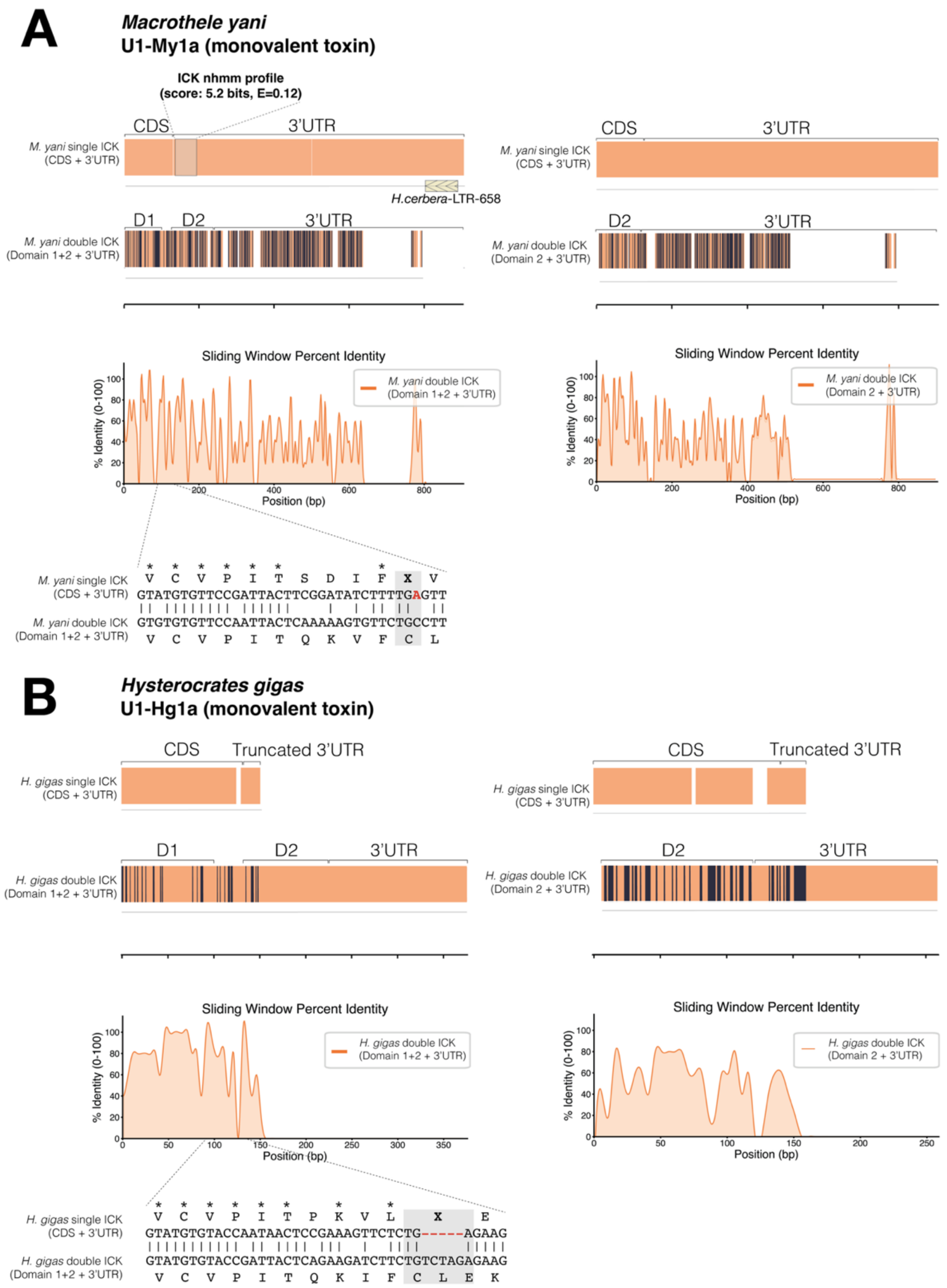

SI Figure S14: Nucleotide alignments of mature peptides and UTRs. Sequence alignment of monovalent mature coding sequence (CDS) and 3'UTR (orange) vs. bivalent mature CDS and 3'UTR (left) and only the second domain and 3'UTR of closest bivalent paralog (right). Identical sites in the bivalent paralog are shown in orange, mismatches in black. TE annotations are shown below each sequence along a grey line, retrotransposons are coloured in yellow, DNA transposons in blue, and direction of insertions indicated by arrows. Corresponding percent sequence identity (%) to the monovalent reference sequence within sliding windows of 5 bp is shown underneath each alignment. The stop codon region in the monovalent sequence aligned to its bivalent paralog is highlighted below (asterisks: identical residue, vertical line: identical nucleotide position, stop codons are coloured in red and highlighted in grey shades. **A)** U1-My1a vs. U1-My2b. **B)** U1-Hg1a vs. U1-Hg2a.

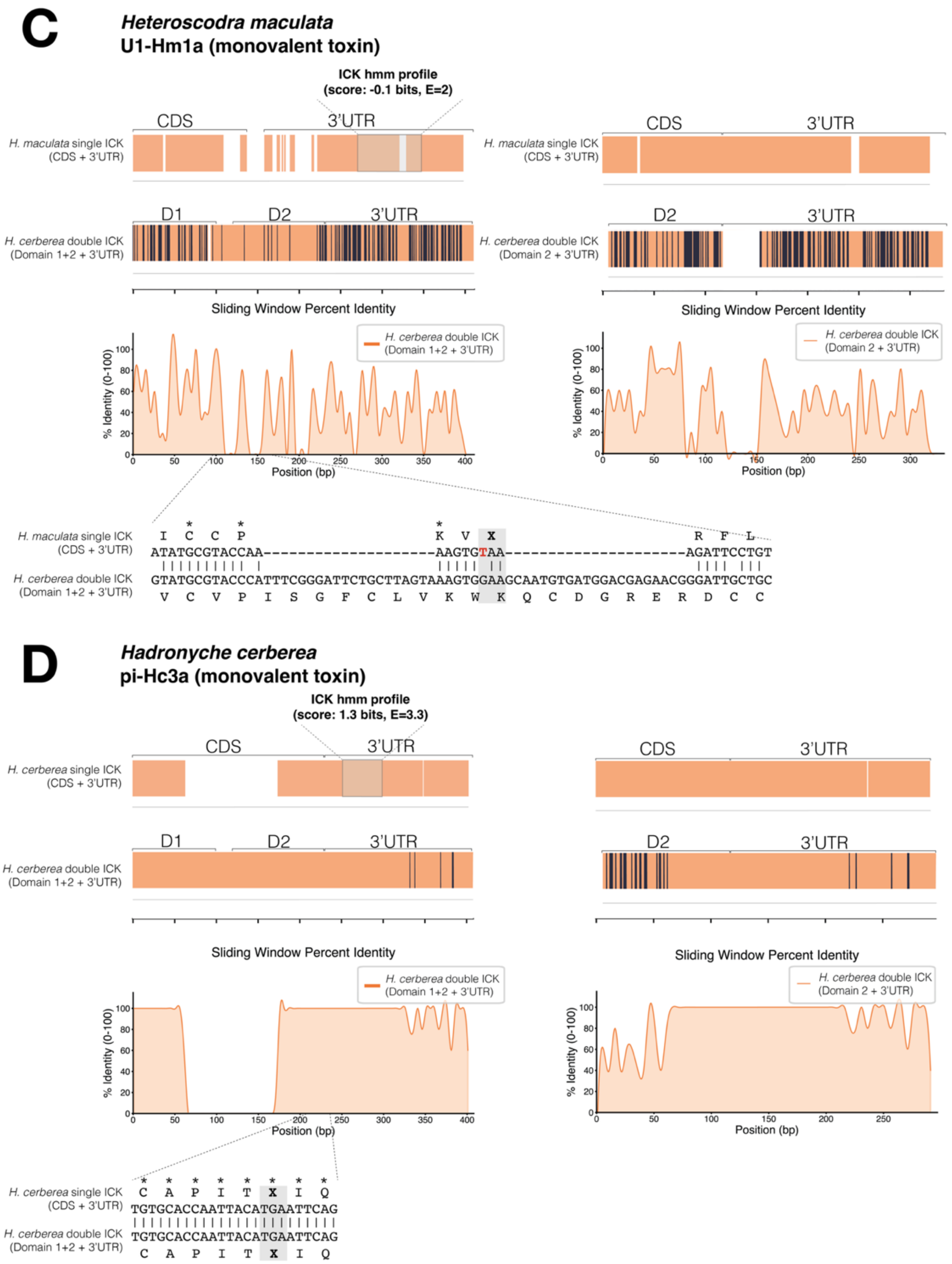

SI Figure S14 (continued): Nucleotide alignments of mature peptides and UTRs. Sequence alignment of monovalent mature coding sequence (CDS) and 3'UTR (orange) vs. bivalent mature CDS and 3'UTR (left) and only the second domain and 3'UTR of closest bivalent paralog (right). Identical sites in the bivalent paralog are shown in orange, mismatches in black. TE annotations are shown below each sequence along a grey line, retrotransposons are coloured in yellow, DNA transposons in blue, and direction of insertions indicated by arrows. Corresponding percent sequence identity (%) to the monovalent reference sequence within sliding windows of 5 bp is shown underneath each alignment. The stop codon region in the monovalent sequence aligned to its bivalent paralog is highlighted below (asterisks: identical residue, vertical line: identical nucleotide position, stop codons are coloured in red and highlighted in grey shades. **A)** U1-Hm1a vs. pi-Hc1a. **B)** pi-Hc3a vs. pi-Hc1a.

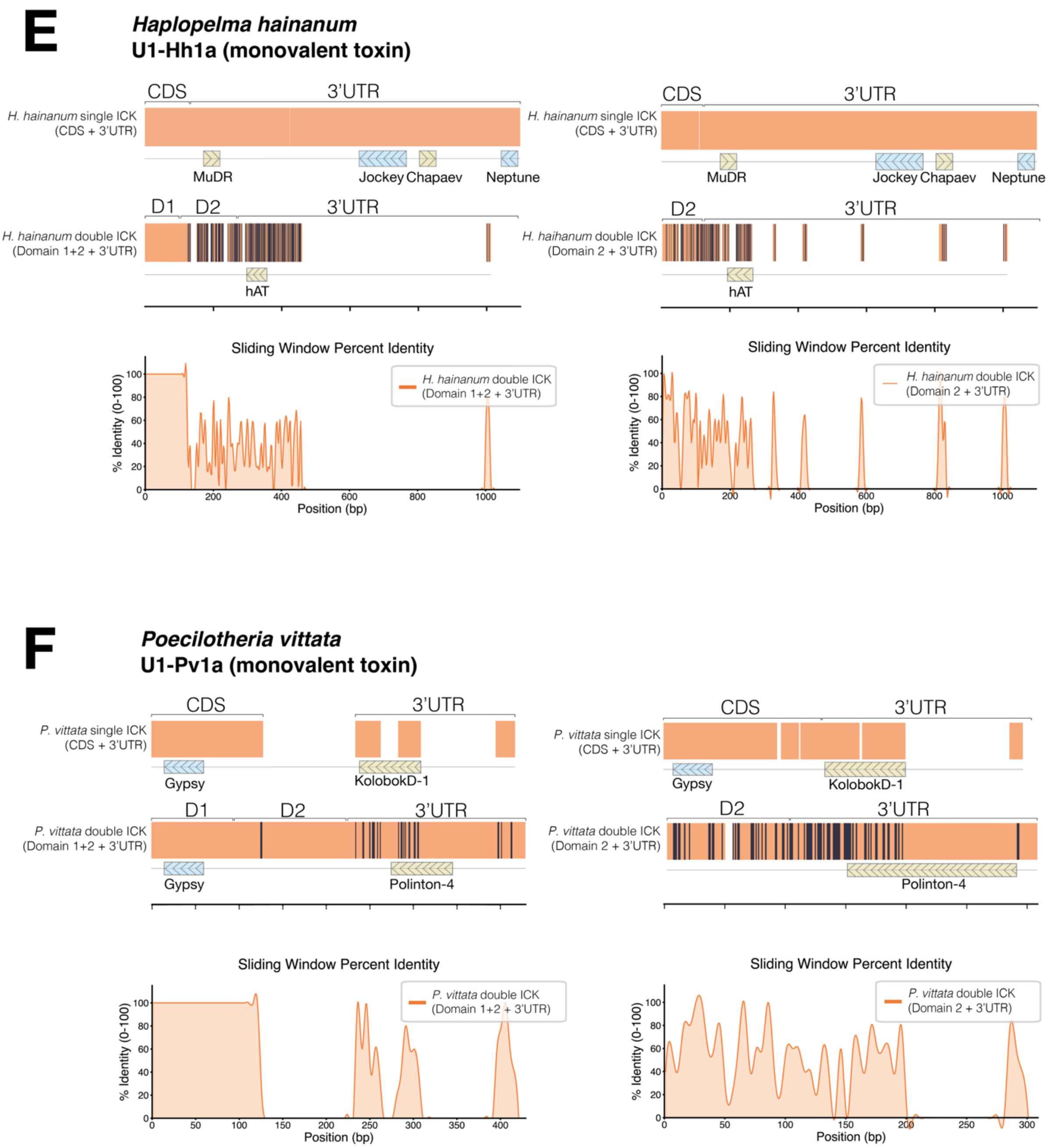

SI Figure S14 (continued): Nucleotide alignments of mature peptides and UTRs. Sequence alignment of monovalent mature coding sequence (CDS) and 3'UTR (orange) vs. bivalent mature CDS and 3'UTR (left) and only the second domain and 3'UTR of closest bivalent paralog (right). Identical sites in the bivalent paralog are shown in orange, mismatches in black. TE annotations are shown below each sequence along a grey line, retrotransposons are coloured in yellow, DNA transposons in blue, and direction of insertions indicated by arrows. Corresponding percent sequence identity (%) to the monovalent reference sequence within sliding windows of 5 bp is shown underneath each alignment. The stop codon region in the monovalent sequence aligned to its bivalent paralog is highlighted below (asterisks: identical residue, vertical line: identical nucleotide position, stop codons are coloured in red and highlighted in grey shades. **A)** U1-Hh1a vs. U1-Hh2a. **B)** U1-Pv1a vs. U1-Pv2a.

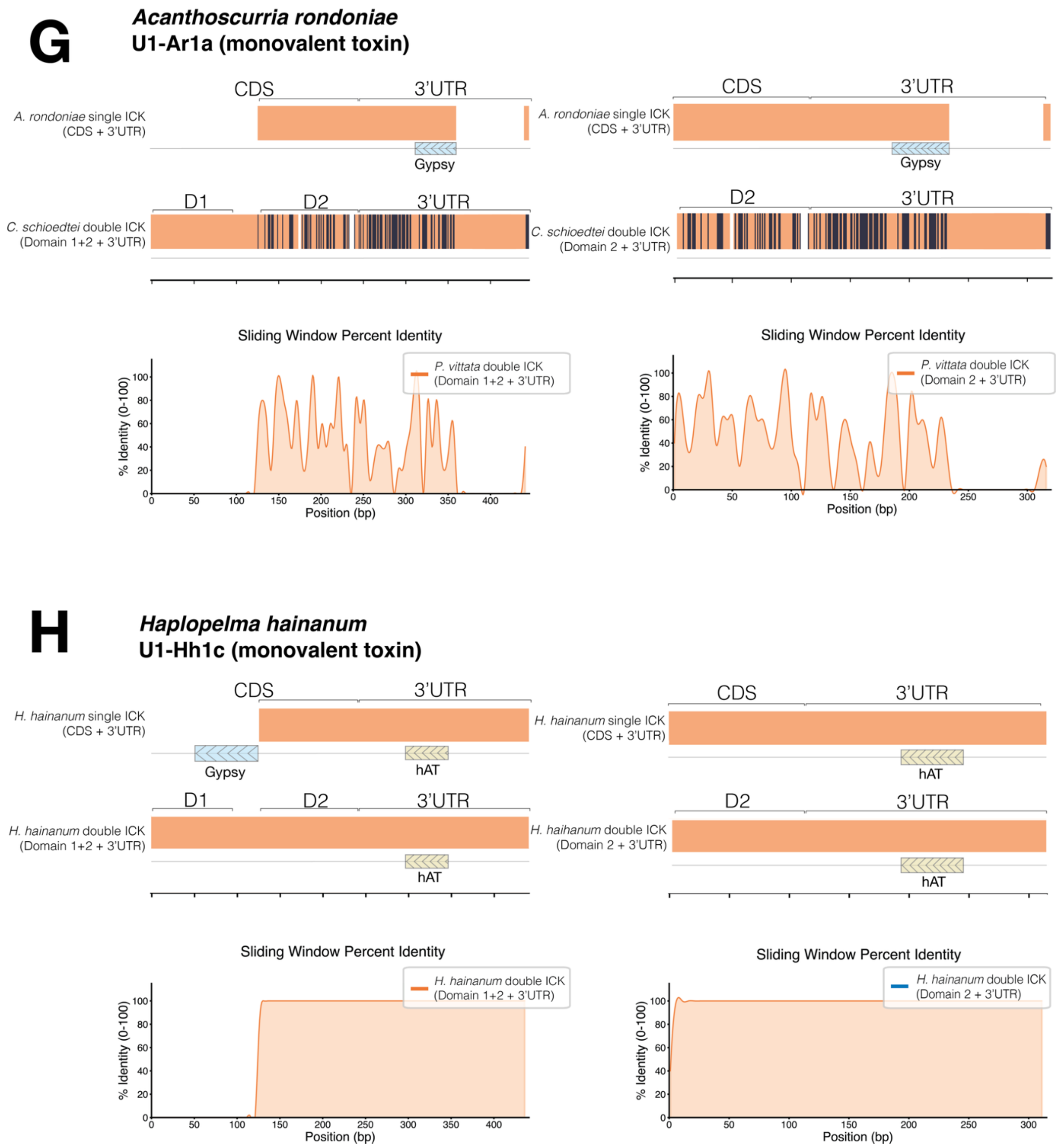

SI Figure S14 (continued): Nucleotide alignments of mature peptides and UTRs. Sequence alignment of monovalent mature coding sequence (CDS) and 3'UTR (orange) vs. bivalent mature CDS and 3'UTR (left) and only the second domain and 3'UTR of closest bivalent paralog (right). Identical sites in the bivalent paralog are shown in orange, mismatches in black. TE annotations are shown below each sequence along a grey line, retrotransposons are coloured in yellow, DNA transposons in blue, and direction of insertions indicated by arrows. Corresponding percent sequence identity (%) to the monovalent reference sequence within sliding windows of 5 bp is shown underneath each alignment. The stop codon region in the monovalent sequence aligned to its bivalent paralog is highlighted below (asterisks: identical residue, vertical line: identical nucleotide position, stop codons are coloured in red and highlighted in grey shades. **A)** U1-Ar1a vs. U1-Cs1a. **B)** U1-Hh1c vs. U1-Hh2a.

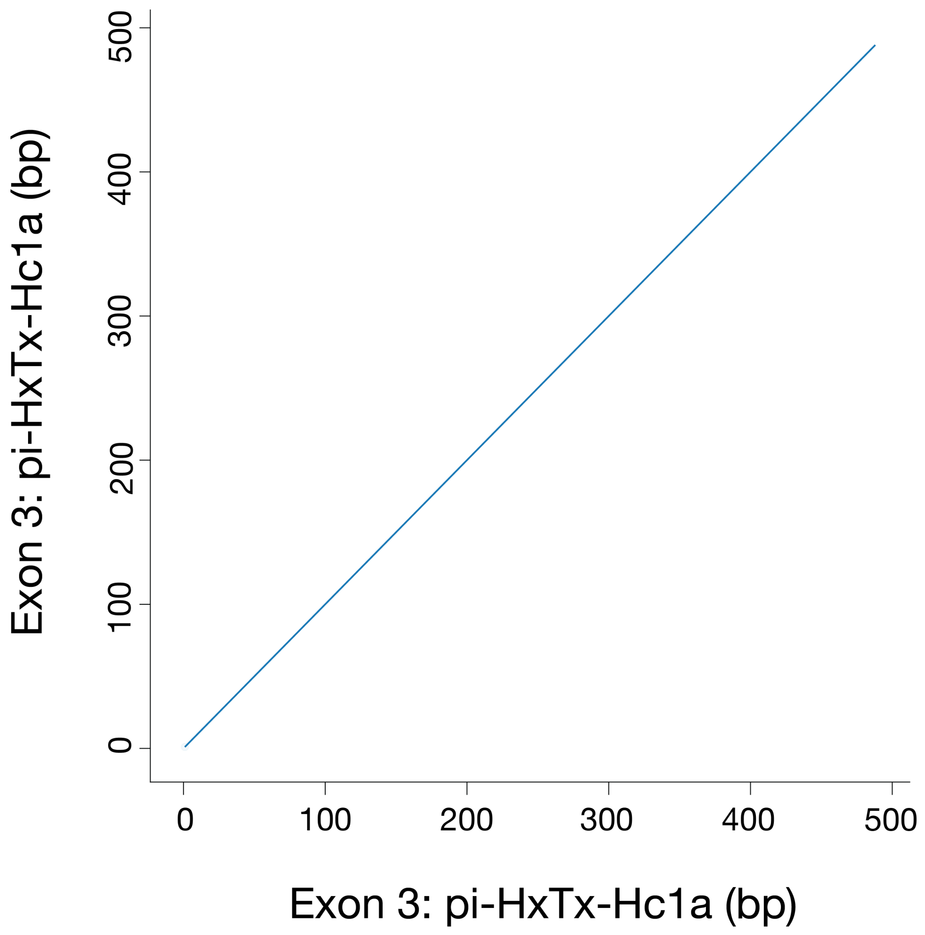

SI Figure S15: Self-dot plot of Hc1a. Self-dot-plotted nucleotide sequence of the third exon of pi-Hexatoxin-Hc1a encoding the mature bivalent peptide. Positions along the x- and y-axis display nucleotide positions, and the dot-plot is shown as a blue line, indicating linearity across the entire CDS.

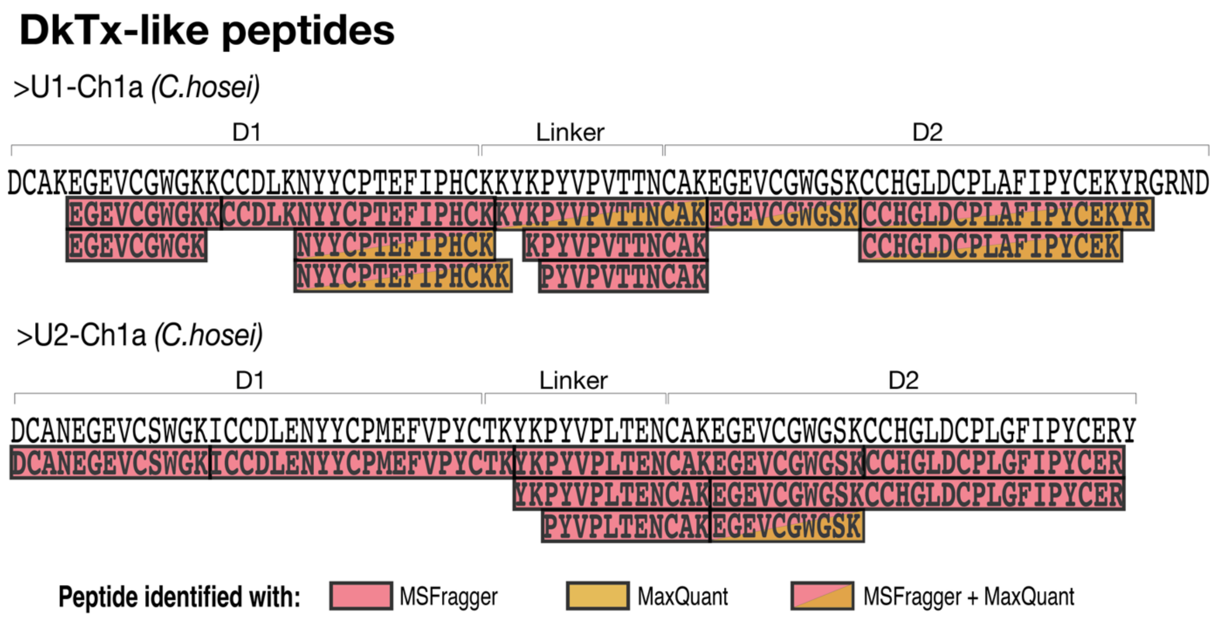

SI Figure S16: Mass spectrometry-identification of DkTx-like peptides. Alignment of tryptic peptide fragments to DkTx-like toxins in *C. hosei*. Peptides are coloured according to the software that was used to identify peptide spectrum matches (red: MSFragger only, yellow: MaxQuant only, red and yellow: identification by both software).

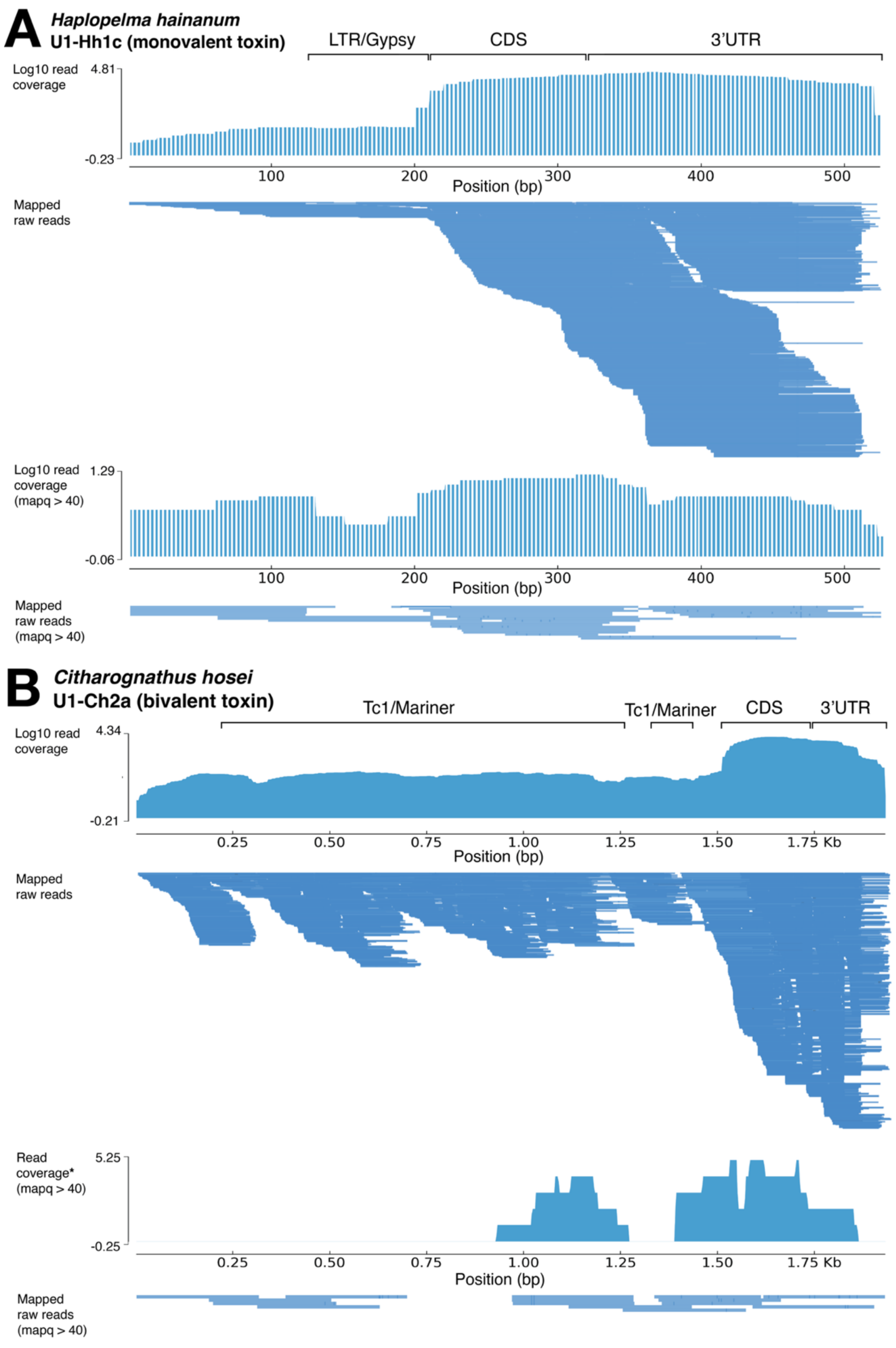

SI Figure S17: Mapping of RNAseq raw reads to RNA transcriptome assemblies. Raw RNA reads from **A)** *H. hainanum* (SRA accession: SRR18936171) and **B)** *C. hosei* mapped to their respective transcriptome assemblies. For each transcript, log10 read coverage within 10 bp windows is displayed across the transcript assembly sequences for unfiltered and filtered (map quality > 40) mapped reads, with the mapped raw reads shown below the coverage tracks as blue lines. Positions on U1-Hh1c is shown in base pairs, and U1-Ch2a in kilobases. *Mapped reads onto U1-Ch2a with map quality > 40 have too low coverage to be log-transformed and is shown as linear values.

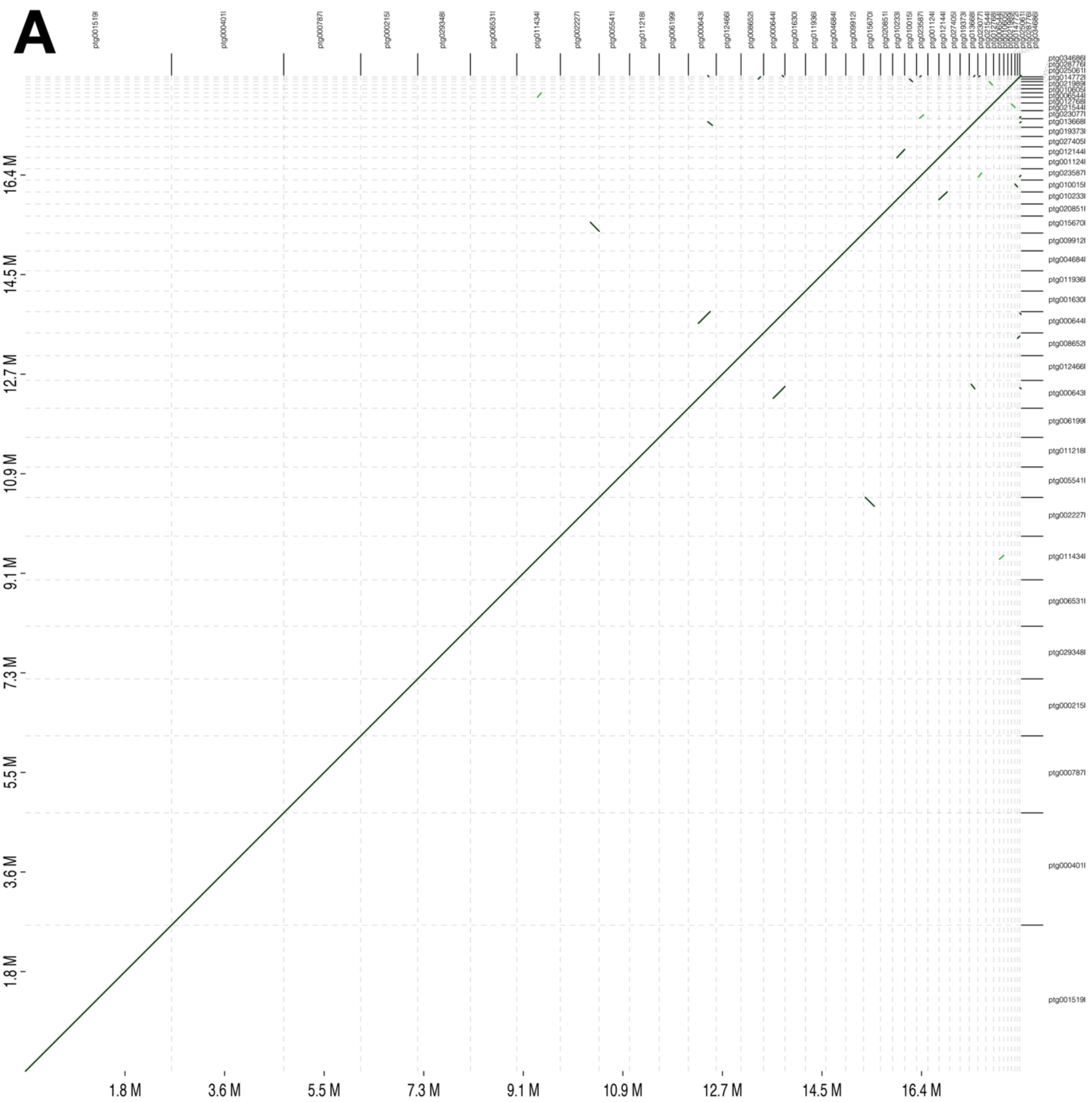

SI Figure S18: Dot-plot of contigs with ICK-encoding genes. All vs. all dot-plot of contigs with annotated ICK-genes using minimap2 (k=19). **A)** Dot-plots of the initial set of 39 contigs with ICK-genes, sorted from largest to smallest. Positions are shown in Mb.

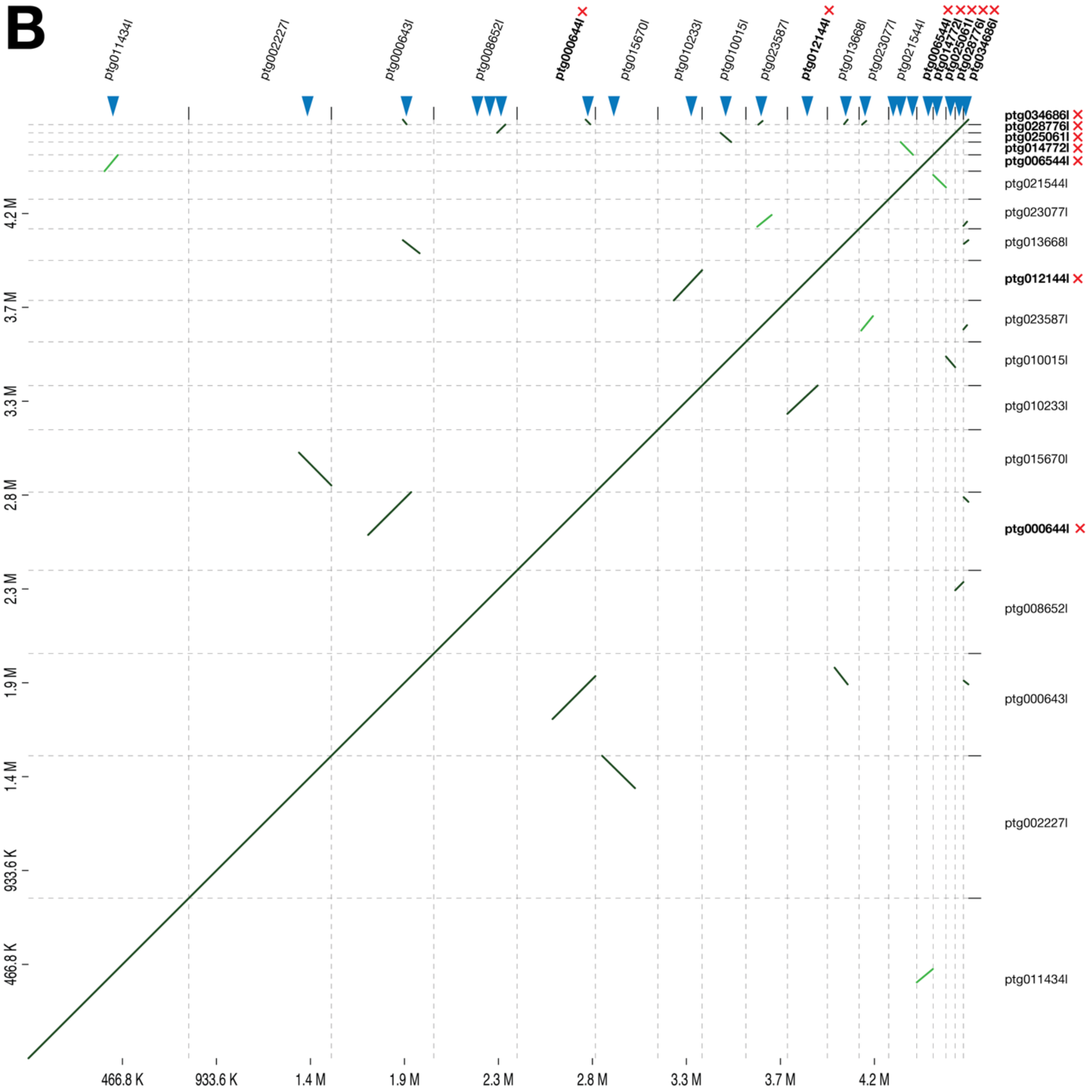

SI Figure S18 (continued): Dot-plot of contigs with ICK-encoding genes. All vs. all dot-plot of contigs with annotated ICK-genes using minimap2 (k=19). **B)** Only displaying contigs with secondary hits with chaining scores >0.5. Approximate ICK gene positions are indicated with blue triangles. Contigs that were subsequently filtered out are highlighted in bold, marked with a red x.

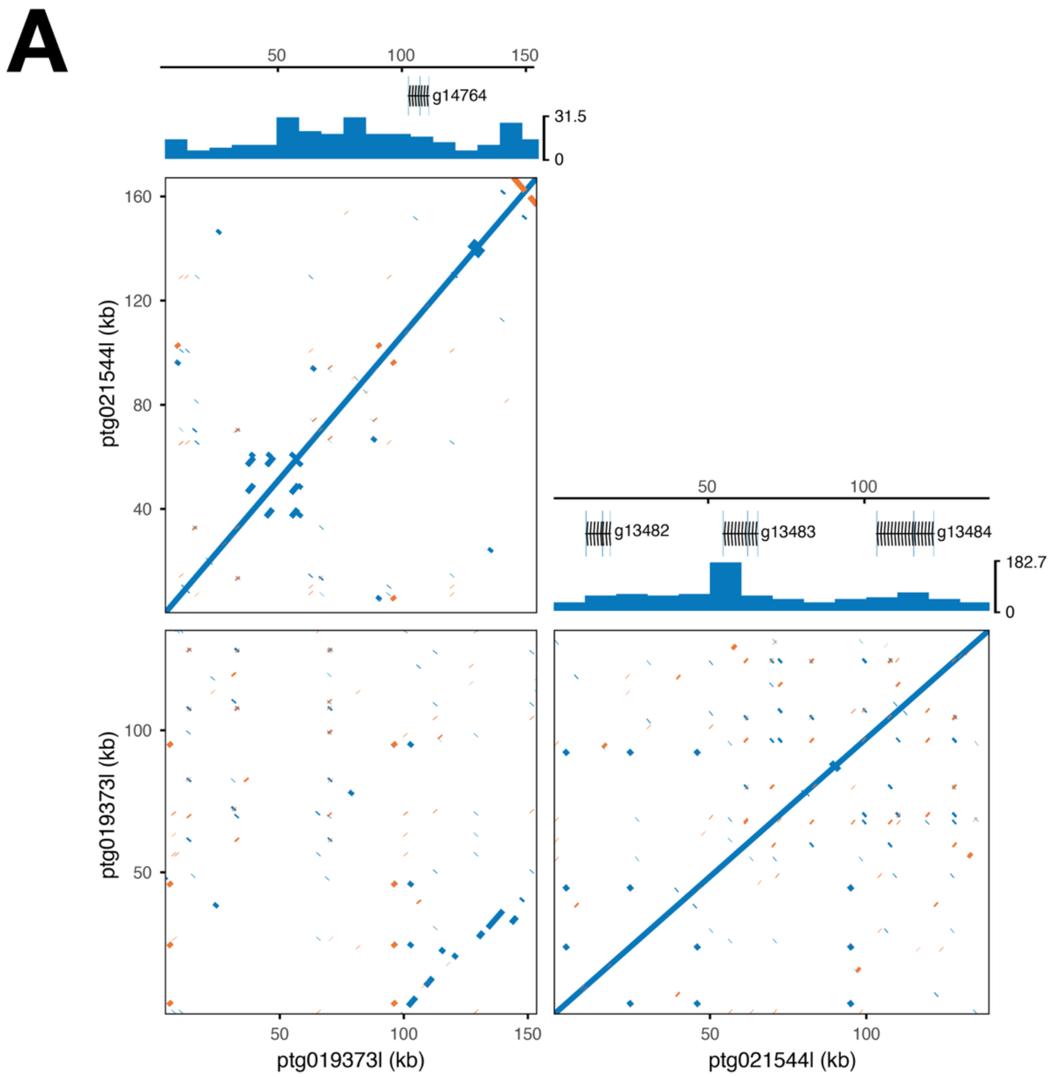

SI Figure S19: Evaluation of contigs with near-identical ICK-encoding genes. Dot-plots of k-mer comparisons (k=15) between contigs harbouring ICK-encoding genes that cluster by >95% identity in their coding sequences (see **SI Table S7**). Contigs length is shown in kb, blue lines are alignments in the forwards direction, orange are reverse. HiFi read coverage along the contigs (per 10 kb) is displayed above each contig (blue), and ICK-encoding genes with gene names are shown on top (exons shown in blue, introns as black arrows showing the direction of the gene). **A)** Comparison of contigs ptg019373l and ptg021544l.

B

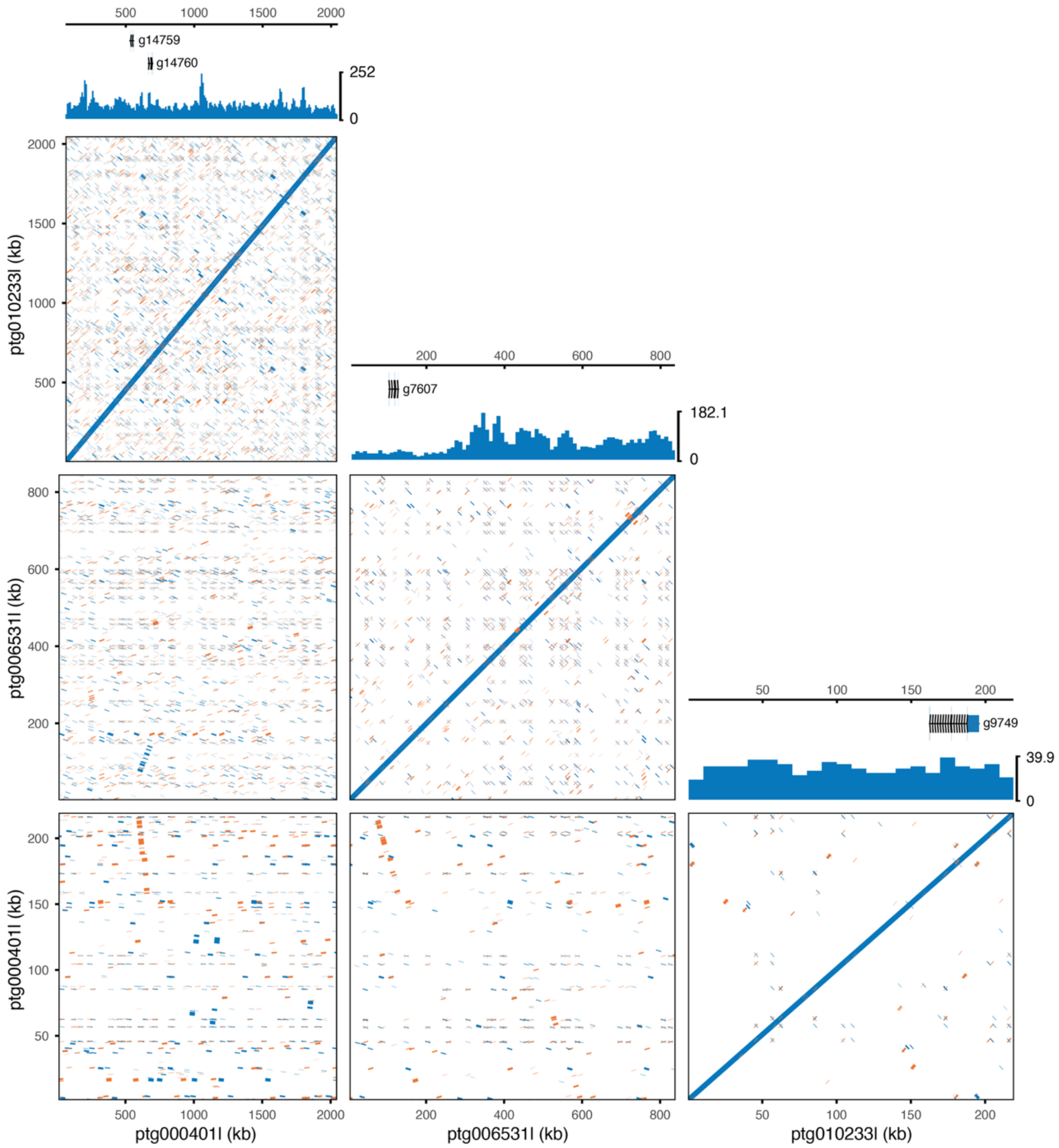

SI Figure S19 (continued): Evaluation of contigs with near-identical ICK-encoding genes. Dot-plots of k-mer comparisons (k=15) between contigs harbouring ICK-encoding genes that cluster by >95% identity in their coding sequences (see **SI Table S7**). Contigs length is shown in kb, blue lines are alignments in the forwards direction, orange are reverse. HiFi read coverage along the contigs (per 10 kb) is displayed above each contig (blue), and ICK-encoding genes with gene names are shown on top (exons shown in blue, introns as black arrows showing the direction of the gene). **B)** Comparison of contigs ptg000401l, ptg006531l and ptg010233l.

C

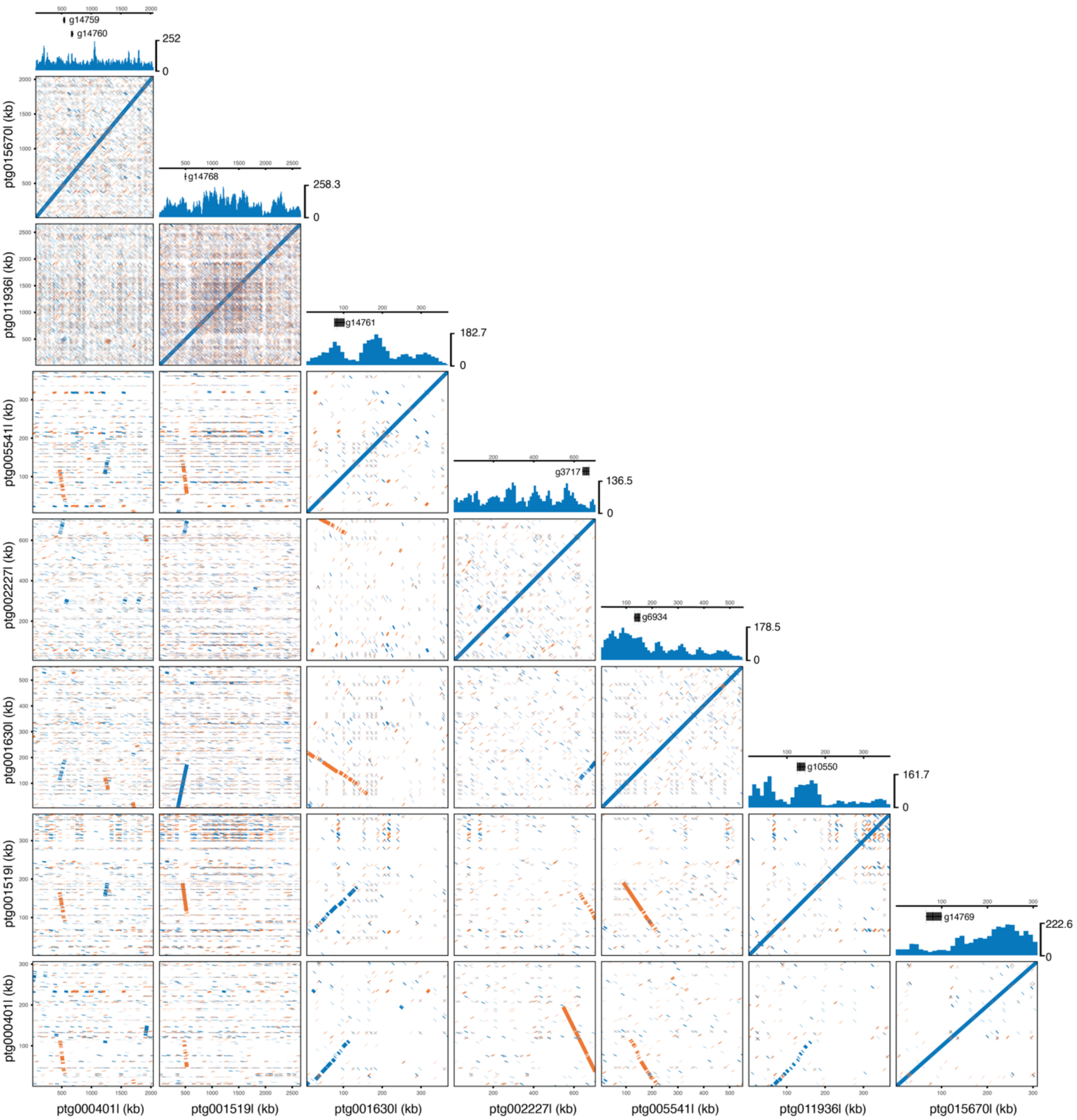

SI Figure S19 (continued): Evaluation of contigs with near-identical ICK-encoding genes. Dot-plots of k-mer comparisons (k=15) between contigs harbouring ICK-encoding genes that cluster by >95% identity in their coding sequences (see **SI Table S7**). Contigs length is shown in kb, blue lines are alignments in the forwards direction, orange are reverse. HiFi read coverage along the contigs (per 10 kb) is displayed above each contig (blue), and ICK-encoding genes with gene names are shown on top (exons shown in blue, introns as black arrows showing the direction of the gene). **C)** Comparison of contigs ptg000401l, ptg001519l, ptg001630l, ptg002227l, ptg005541l, ptg011936l, and ptg015670l.

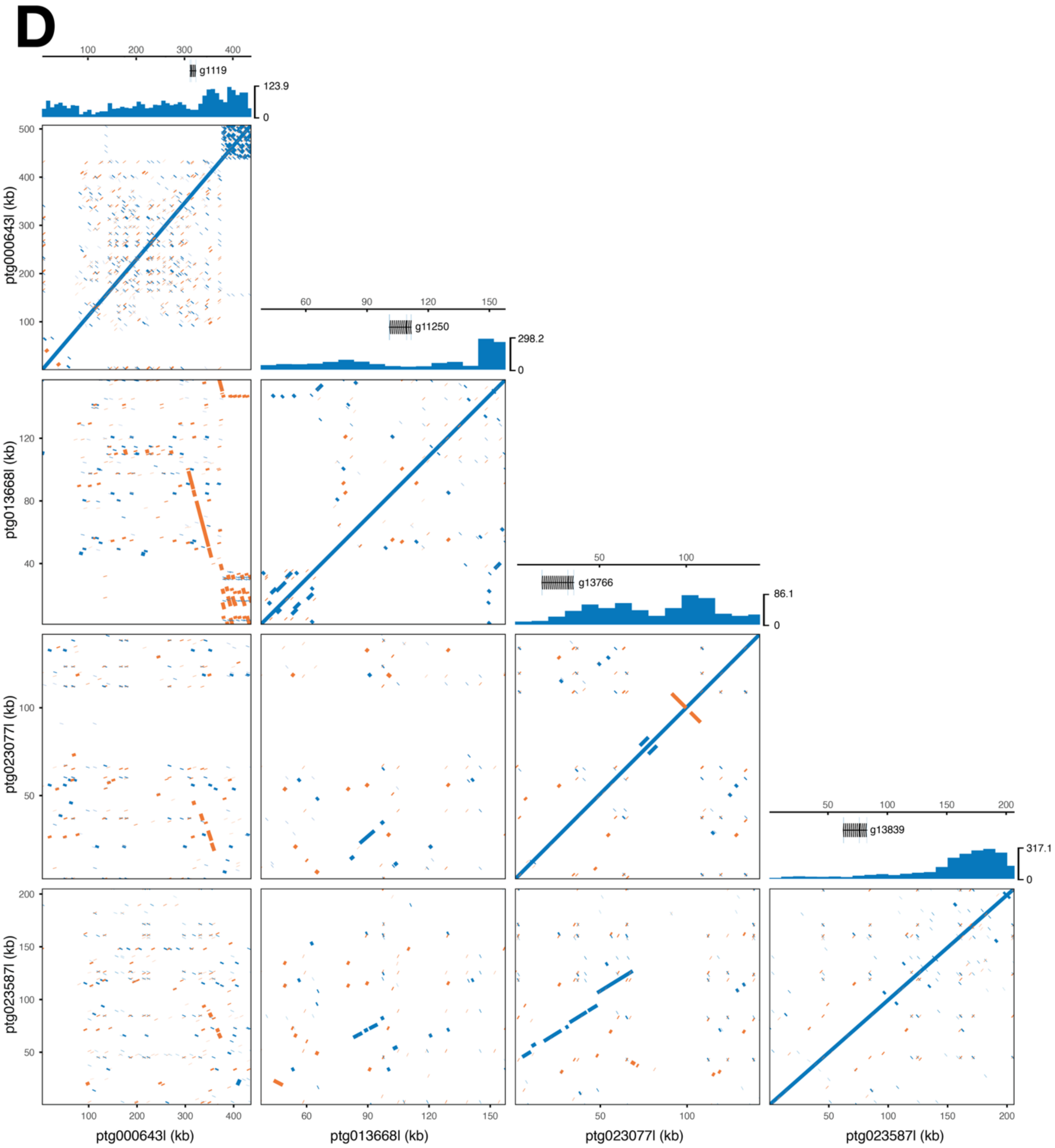

SI Figure S19 (continued): Evaluation of contigs with near-identical ICK-encoding genes. Dot-plots of k-mer comparisons (k=15) between contigs harbouring ICK-encoding genes that cluster by >95% identity in their coding sequences (see **SI Table S7**). Contigs length is shown in kb, blue lines are alignments in the forwards direction, orange are reverse. HiFi read coverage along the contigs (per 10 kb) is displayed above each contig (blue), and ICK-encoding genes with gene names are shown on top (exons shown in blue, introns as black arrows showing the direction of the gene). **D)** Comparison of contigs ptg000643l, ptg013668, ptg023077l, and ptg023587l.

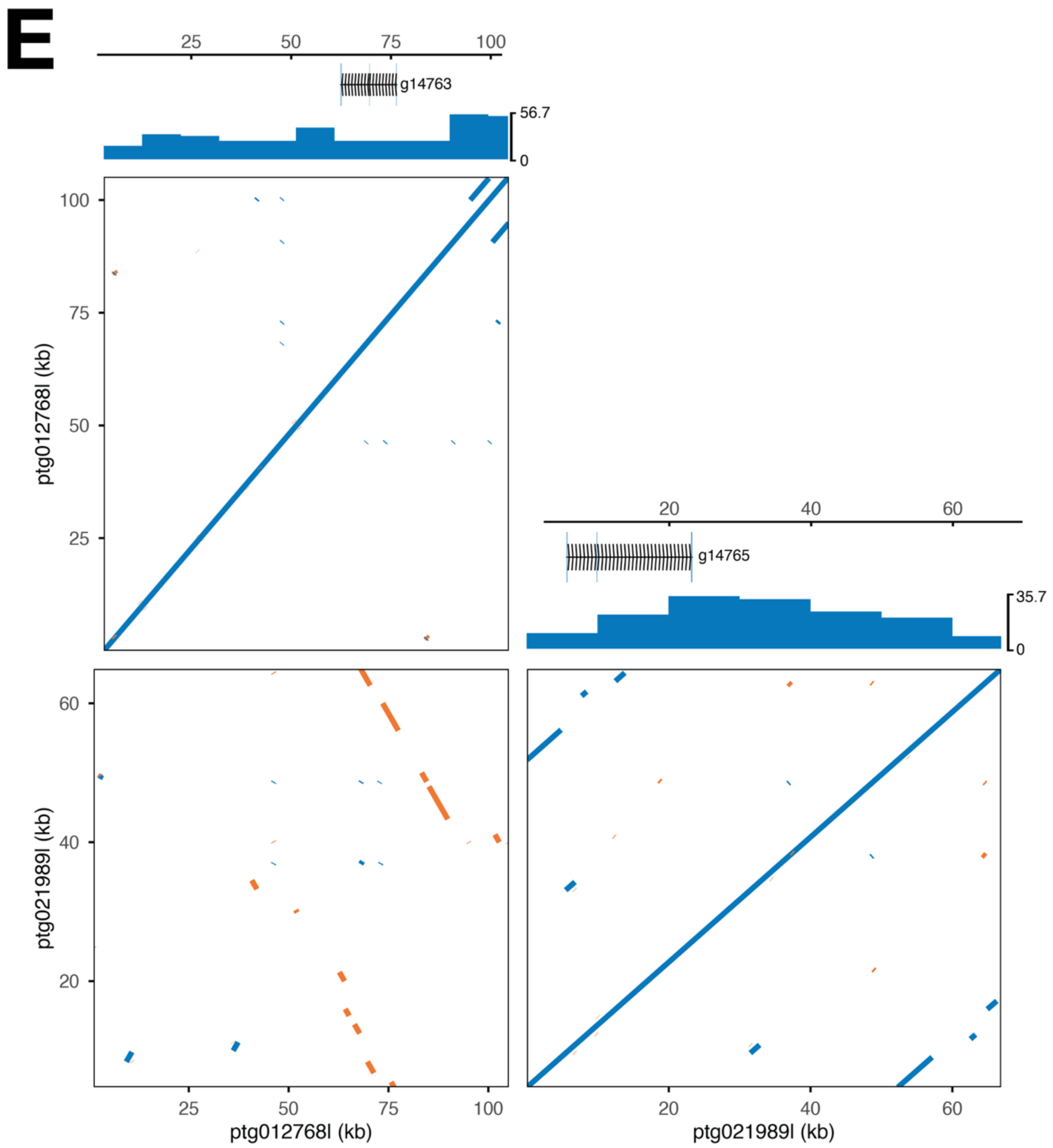

SI Figure S19 (continued): Evaluation of contigs with near-identical ICK-encoding genes. Dot-plots of k-mer comparisons (k=15) between contigs harbouring ICK-encoding genes that cluster by >95% identity in their coding sequences (see **SI Table S7**). Contigs length is shown in kb, blue lines are alignments in the forwards direction, orange are reverse. HiFi read coverage along the contigs (per 10 kb) is displayed above each contig (blue), and ICK-encoding genes with gene names are shown on top (exons shown in blue, introns as black arrows showing the direction of the gene). **E)** Comparison of contigs ptg012768l and ptg021989l.

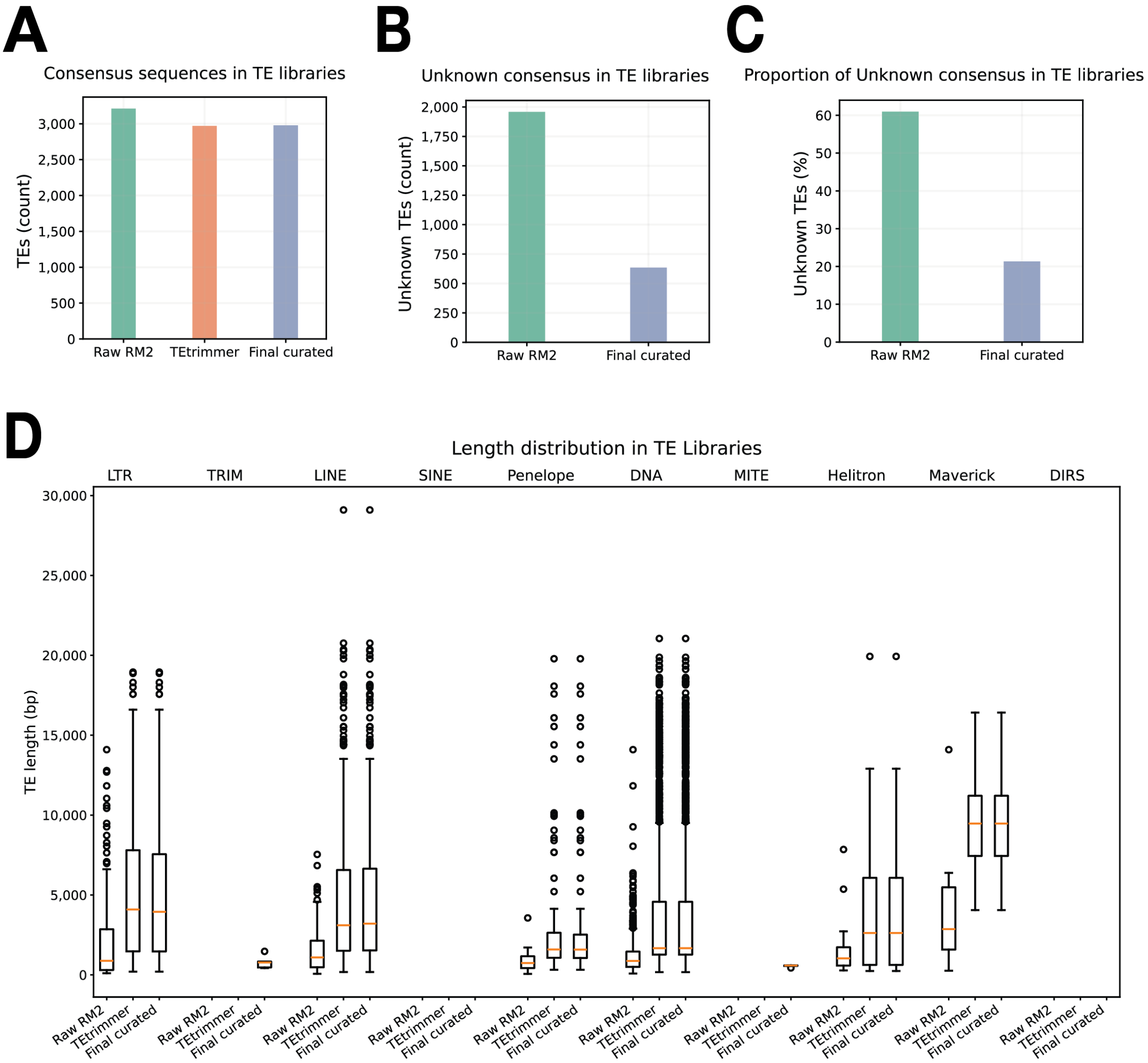

SI Figure S20: Comparison of transposable element libraries. A) Number of consensus sequences in the raw TE library output from RepeatModeler2 (RM2), non-redundant TEtrimmer library, and final curated library. **B)** Number of unclassified (Unknown) consensus sequences in the raw RM2 library and final curated library. **C)** Proportion (%) of unclassified (Unknown) consensus sequences in the raw RM2 library and final curated library. **D)** Length distributions of consensus sequences across different TE orders in the RM2, TEtrimmer, and final curated TE libraries.
